# LSD alters dynamic integration and segregation in the human brain

**DOI:** 10.1016/j.neuroimage.2020.117653

**Published:** 2021-02-15

**Authors:** Andrea I. Luppi, Robin L. Carhart-Harris, Leor Roseman, Ioannis Pappas, David K. Menon, Emmanuel A. Stamatakis

**Affiliations:** a Division of Anaesthesia, School of Clinical Medicine, University of Cambridge, Cambridge CB2 0QQ, United Kingdom; b Department of Clinical Neurosciences, University of Cambridge, Cambridge CB2 0QQ, United Kingdom; c Center for Psychedelic Research, Department of Brain Science, Imperial College London, London W12 0NN, United Kingdom; d Wolfson Brain Imaging Centre, University of Cambridge, Cambridge CB2 0QQ, United Kingdom

**Keywords:** LSD, Integration-segregation, FMRI dynamics, Complexity, Structure-function, Small-world network

## Abstract

Investigating changes in brain function induced by mind-altering substances such as LSD is a powerful method for interrogating and understanding how mind interfaces with brain, by connecting novel psychological phenomena with their neurobiological correlates. LSD is known to increase measures of brain complexity, potentially reflecting a neurobiological correlate of the especially rich phenomenological content of psychedelic-induced experiences. Yet although the subjective stream of consciousness is a constant ebb and flow, no studies to date have investigated how LSD influences the dynamics of functional connectivity in the human brain. Focusing on the two fundamental network properties of integration and segregation, here we combined graph theory and dynamic functional connectivity from resting-state functional MRI to examine time-resolved effects of LSD on brain networks properties and subjective experiences. Our main finding is that the effects of LSD on brain function and subjective experience are non-uniform in time: LSD makes globally segregated sub-states of dynamic functional connectivity more complex, and weakens the relationship between functional and anatomical connectivity. On a regional level, LSD reduces functional connectivity of the anterior medial prefrontal cortex, specifically during states of high segregation. Time-specific effects were correlated with different aspects of subjective experiences; in particular, ego dissolution was predicted by increased small-world organisation during a state of high global integration. These results reveal a more nuanced, temporally-specific picture of altered brain connectivity and complexity under psychedelics than has previously been reported.

## Highlights

• LSD untethers functional connectivity from the constraint of structural connectivity.
• Increased small-worldness of brain networks predicts LSD-induced ego-dissolution.
• LSD has time-specific effects on brain network integration and segregation.
• LSD increases the complexity of segregated brain states.
• Results bridge pharmacodynamics, subjective experience and brain dynamics.

## 1 Introduction

The classic serotonergic psychedelic, LSD, induces a profoundly altered state of consciousness, the neural correlates of which are just beginning to be unravelled (Carhart-Harris et al., 2016). Combining pharmacological interventions with non-invasive brain imaging techniques such as functional MRI, affords the dual advantage of improving our understanding of a potent psychoactive drug's effects, while providing insight into normal and abnormal brain function. In recent years, relevant studies have been carried out with classic 'psychedelic' (‘mind-manifesting’) drugs, which all share agonist properties at the serotonin 5-HT_2A_ receptor. Examples of classic 5-HT_2A_ receptor agonist psychedelics include the synthetic compound lysergic acid diethylamide (LSD), the Psilocybe mushroom constituent psilocybin, and dimethyltryptamine (DMT), an ingredient of the ritual beverage ayahuasca (dos Santos et al., 2016).

Moreover, evidence has been accumulating for the therapeutic potential of carefully administered psychedelics for improving mental health outcomes (Johnson et al., 2019; Liechti, 2017; Nichols, 2016), highlighting the need for further research into their brain effects. Especially intriguing is the observation of increased brain complexity during the psychedelic experience induced by psilocybin, LSD and DMT (Lebedev et al., 2016; Tagliazucchi et al., 2014; Viol et al., 2017; Varley et al., 2020), which has motivated the so-called ‘Entropic Brain’ and more recent ‘Anarchic Brain’ Hypotheses (Carhart-Harris, 2018; Carhart-Harris et al., 2014; Carhart-Harris and Friston, 2019), according to which measures of brain complexity (such as entropy and Lempel-Ziv compressibility) are said to reflect the diversity or 'richness' of one's subjective experiences or content of consciousness. Evidence of decreased brain complexity when consciousness is diminished or lost, for instance during deep sleep or anaesthesia (Varley et al., 2020; Burioka et al., 2005; Liu et al., 2019; Olofsen et al., 2008; Schartner et al., 2015), further supports the entropic brain principle. Thus, brain complexity may offer a proficuous way of indexing the psychological effects of mind-altering substances via their neurobiological effects.

Nevertheless, the subjective stream of consciousness is a constant ebb and flow – yet only recently have investigations of altered states of consciousness begun to take brain dynamics into account (Atasoy et al., 2018, 2017; Deco et al., 2018; Kringelbach et al., 2020; Lord et al., 2019), grounded in the emerging understanding that spatio-temporal dynamics may represent the “common currency” between the mind (subjective phenomenology) and the underlying neurobiology (Carhart-Harris et al., 2014; Northoff et al., 2020). Indeed, recent studies using dynamic functional connectivity (dFC) in the healthy brain have demonstrated that patterns of brain connectivity are not stationary, but rather vary over time, switching between different dynamic sub-states with different relevance for cognition (Allen et al., 2014; Demertzi et al., 2019; Fukushima et al., 2018; Lurie et al., 2018; Preti et al., 2017; Shine et al., 2016)^,^

In particular, the dynamics of brain integration and segregation are increasingly recognised to be important properties of the mind and brain, relevant to our understanding of conscious experience. Subjectively, humans experience the world as an amalgamation (integration) of distinct sensory streams (segregation) (Tononi, 2004). Neurobiologically, information processed in parallel by domain-specific systems must be brought together and integrated, in order to guide adaptive behaviour (Dehaene et al., 2011; Dehaene and Changeux, 2011; Luppi et al., 2020). Evidence from previous neuroimaging studies indicates that LSD and other psychedelics, such as psilocybin, decrease the integrity of functional connectivity (FC) within resting-state networks (RSNs), but increase FC between normally distinct RSNs (Carhart-Harris et al., 2016; Johnson et al., 2019; Müller et al., 2018; Roseman et al., 2014). Such effects are typically interpreted as representing increased global-integration and decreased global-segregation in the brain (dos Santos et al., 2016). However, a formal characterisation of their dynamics across time is still missing. Additionally, dynamic sub-states dominated by integration or segregation have recently been found to have different relevance for consciousness, e.g. in patients with disorders of consciousness and propofol anaesthesia, with the predominantly integrated state showing shared reductions in complexity when consciousness is lost (Luppi et al., 2019).

These observations raise the intriguing possibility that the potent effects of LSD on the human brain and mind, may also have a hitherto unexplored finer-grained temporal dimension, in terms of brain integration and segregation, and thus neglecting this would represent a scientific oversight and underutilisation. Here, we endeavour to address this question.

By representing the brain as a network of nodes (brain regions) connected by edges (estimated by the strength of functional connectivity between regions), graph theory provides a formal way to precisely quantify integration, segregation and their dynamics in the brain. Substantial recent progress in our understanding of human brain function has derived from studying the brain as a complex network (Bullmore and Sporns, 2009; Sporns, 2011). A crucial property of many natural and artificial networks, including the human brain, is the so-called ‘small-world’ organisation (Bassett and Bullmore, 2017). A small-world network combines the elevated clustering coefficient typical of lattice networks (theorised to support specialised processing) with the short characteristic path length typica of random networks (facilitating integration between different clusters). Thus, small-world organisation may represent a mark of optimal balance between global and local processing (Bassett and Bullmore, 2017) – and indeed, previous research indicates that small-world character is diminished in a dynamic fashion during loss of consciousness induced by anaesthesia or brain injury (Barttfeld et al., 2015; Luppi et al., 2019).

We therefore combined graph theory with dynamic functional MRI connectivity to explore the time-resolved effects of LSD on brain network organisation and function, in a placebo-controlled study with 15 healthy volunteers with previous psychedelic experience. Our main objective was to establish whether the effects of LSD on brain function and the ensuing subjective alterations of consciousness are temporally diverse or uniform. Following the Entropic/Anarchic brain framework, we further hypothesised that the effects of LSD on dynamic brain states should be broadly opposite to those observed during loss of consciousness (Barttfeld et al., 2015; Luppi et al., 2019).

## 2 Materials and methods

### 2.1 Data acquisition

#### 2.1.1 Ethics statement

This study was approved by the National Research Ethics Service Committee London–West London and was conducted in accordance with the revised Declaration of Helsinki (2000), the International Committee on Harmonization Good Clinical Practice guidelines and National Health Service Research Governance Framework. Imperial College London sponsored the research, which was conducted under a Home Office license for research with schedule 1 drugs.

#### 2.1.2 Recruitment

The data acquisition protocols were described in detail in a previous paper (Carhart-Harris et al., 2016), so we will describe them in brief here. Twenty healthy volunteers with a previous experience using psychedelic drugs were scanned. Patients underwent two scans, 14 days apart. On one day they were given a placebo (10-mL saline) and the other they were given an active dose of LSD (75 μg of LSD in 10-mL saline). The infusion (drug/placebo) was administered over 2 minutes and occurred 115min before the resting-state scans were initiated. After infusion, subjects had a brief acclimation period in a mock MRI scanner to prepare them for the experience of being in the real machine. ASL and BOLD scanning consisted of three seven-minute eyes closed resting state scans.

The first and third scans were eyes-closed, resting state without stimulation, while the second scan involved listening to music; however, this scan was not used in this analysis, as the focus was on the dynamics of resting-state functional connectivity in the absence of exogenous stimulation. After each seven-minute scan, VAS (visual analog scale) ratings were performed in the scanner via a response-box (Supplementary Materials and Methods). At the end of scanning days, participants also completed the 11-item Altered States of Consciousness (ASC) scale (Studerus et al., 2010), providing retrospective subjective evaluations pertaining to the peak of the experience (i.e. during fMRI scanning) (Carhart-Harris et al., 2016).

The precise length of each of the two BOLD scans included here was 7:20 minutes. Imaging was performed on a 3T GE HDx system. High-resolution anatomical images were acquired with 3D fast spoiled gradient echo scans in an axial orientation, with field of view = 256 × 256 × 192 and matrix = 256 × 256 × 129 to yield 1mm isotropic voxel resolution. TR/TE = 7.9/3.0ms; inversion time = 450ms; flip angle = 20.

BOLD-weighted fMRI data were acquired using a gradient echo planer imaging sequence, TR/TE = 2000/35ms, FoV = 220mm, 64 × 64 acquisition matrix, parallel acceleration factor = 2, 90 flip angle. Thirty-five oblique axial slices were acquired in an interleaved fashion, each 3.4mm thick with zero slice gap (3.4mm isotropic voxels). One subject aborted the experiment due to anxiety and four others we excluded for excessive head motion in the scanner (defined as > 15% of volumes with mean frame-wise displacement > 0.5 (Carhart-Harris et al., 2016)), leaving 15 subjects for analysis. Subjective ratings were also obtained from each participant (Supplementary Metarials and Methods).

### 2.2 Preprocessing

We followed the same preprocessing pipeline described in our previous publications (Luppi et al., 2020, 2019): the preprocessing and image analysis were performed using the CONN toolbox, version 17f (CONN; http://www.nitrc.org/projects/conn) (Whitfield-Gabrieli and Nieto-Castanon, 2012) based on Statistical Parametric Mapping 12 (http://www.fil.ion.ucl.ac.uk/spm), implemented in MATLAB. For each condition (placebo and LSD) we applied a standard preprocessing pipeline comprising the following steps: removal of the first three volumes, to eliminate saturation effects and achieve steady-state magnetization; functional realignment to correct for movement; slice-timing correction to account for differences in time of acquisition between slices; identification of outlier scans for subsequent scrubbing by means of the quality assurance/artifact rejection software *art* (http://www.nitrc.org/projects/artifact_detect), adopting the default CONN settings of 5 global signal Z-values and 0.9mm for the identification of outlier volumes; normalisation to Montreal Neurological Institute (MNI-152) standard space with 2mm isotropic resampling resolution, using the segmented grey matter image from each volunteer's high-resolution T1-weighted image, together with an *a priori* grey matter template; spatial smoothing with a Gaussian kernel of 6mm full width at half-maximum (FWHM).

### 2.3 Denoising

Denoising was performed using the CONN toolbox. As for preprocessing, we followed the same denoising described in our previous publications (Luppi et al., 2020, 2019): To reduce noise due to cardiac and motion artifacts, which are known to impact functional connectivity and network analyses (Power et al., 2012; van Dijk et al., 2012), we applied the anatomical CompCor method of denoising the functional data (Behzadi Y et al., 2007), also implemented within the CONN toolbox. The step of global signal regression (GSR) has received substantial attention in the literature (Andellini et al., 2015; Lydon-Staley et al., 2019; Power et al., 2014). Here, we chose to avoid GSR in favour of the aCompCor denoising procedure, in line with previous studies (Carhart-Harris et al., 2016; Luppi et al., 2019). Additionally, here one of our variables of interest is the proportion of anticorrelations between brain regions across different states; however, GSR mathematically mandates that approximately 50% of correlations between regions will be negative (Braun et al., 2012), thereby removing any potentially meaningful differences. The cartographic profile method employed here to identify integrated and segregated sub-states of dynamic functional connectivity (see below) has also been shown to be robust to the use of GSR (Shine et al., 2016).

The aCompCor method involves regressing out of the functional data the following confounding effects: the first five principal components attributable to each individual's white matter signal, and the first five components attributable to individual cerebrospinal fluid (CSF) signal (both computed by applying a one-voxel binary erosion step to the masks of voxels with values above 50% in the corresponding posterior probability maps (Whitfield-Gabrieli and Nieto-Castanon, 2012)); six subject-specific realignment parameters (three translations and three rotations) as well as their first-order temporal derivatives; scrubbing of the outlying scans previously identified by *art* during preprocessing (Placebo: mean = 1.16 ± 1.48% of volumes; LSD: mean = 1.63 ± 2.45% of volumes; max number of scrubbed volumes per scan: 7.4%); and main effect of scanning session. In CONN, confounding factors are removed separately for each voxel and for each subject and session, using Ordinary Least Squares (OLS) regression to project each BOLD signal timeseries to the sub-space orthogonal to all potential confounding effects (Whitfield-Gabrieli and Nieto-Castanon, 2012). Finally, linear detrending was also applied, and the subject-specific denoised BOLD signal timeseries were band-pass filtered to eliminate both low-frequency drift effects and high-frequency noise, thus retaining frequencies between 0.008 and 0.09 Hz.

### 2.4 Functional connectivity analysis

#### 2.4.1 Definition on regions of interest

To construct matrices of functional connectivity, spatially normalised brains were parcellated into 200 cortical regions of interest (ROIs), obtained from the scale-200 version of the recent multi-scale local-global functional parcellation of (Schaefer et al., 2018). Since this parcellation only includes cortical regions, it was augmented with 32 subcortical ROIs from the highest resolution of the recent Melbourne subcortical functional parcellation (Tian et al., 2020). We refer to this composite 232-ROI parcellation as the “augmented Schaefer-232” (Luppi and Stamatakis, 2020).

To ensure the robustness of our analyses to the choice of parcellation, we replicated them using two alternative ways of parcellating the brain. Specifically, robustness to parcellation size was ensured by employing a finer-grained version of the multi-scale Schaefer functional cortical atlas (Schaefer et al., 2018) (with 400 cortical ROIs instead of 200) supplemented with a corresponding finer-grained version of the Melbourne functional subcortical atlas of Tian and colleagues (Luppi and Stamatakis, 2020; Tian et al., 2020) (with 54 subcortical ROIs instead of 32). Furthermore, to ensure robustness to the type of parcellation, we employed the Brainnetome atlas (Fan et al., 2016), which comprises 210 cortical and 36 subcortical ROIs (similar in number to the augmented Schaefer-232), obtained from multimodal (anatomical and functional) connectivity. Results presented in the main text pertain to the Schaefer-232 atlas, with corresponding results for the two alternative parcellations presented in the Supplementary Information.

#### 2.4.2 Connectivity matrix construction

Following our previous work (Luppi et al., 2019), “The time-courses of denoised BOLD signals were averaged between all voxels belonging to a given ROI, using the CONN toolbox. The resulting region-specific time-courses of each subject were then extracted for further analysis in MATLAB version 2016a (http://www.mathworks.co.uk/products/matlab/). Functional connectivity was then estimated as the Pearson correlation coefficient between the time-courses of each pair of ROIs, over the full scanning length”.

#### 2.4.3 Dynamic functional connectivity

Dynamic connectivity matrices were derived using an overlapping sliding-window approach (Allen et al., 2014; Barttfeld et al., 2015), following the same procedure described in previous work (Luppi et al., 2019): For each subject and each condition, BOLD signal data from the first and third scans (resting-state without music) were concatenated along the temporal dimension. Then, tapered sliding windows were obtained by convolving a rectangle of 22 TRs (44s) with a Gaussian kernel of 3 TRs, sliding with 1 TR step size (Allen et al., 2014). The chosen window size of 44s is in the recommended range of 30-60s for capturing spontaneous fluctuations in dFC and obtaining stable network descriptions; tapered sliding windows are also recommended, because they minimise the effects of outlying observations and spurious correlations (Preti et al., 2017). Within each of the resulting overlapping temporal windows of 22 TRs, a 232-by-232 matrix of functional connectivity between ROIs was estimated (or 454-by-454 for the Schaefer-454 atlas, and 246-by-246 for the Brainnetome atlas). Hence, for each condition of each subject we obtained a 3D tensor, consisting of one functional connectivity matrix for each timepoint.

### 2.5 Derivation of Integrated and Segregated sub-states

Following Shine *et al.,* (2016) and subsequent work using the same methodology (Fukushima et al., 2018; Luppi et al., 2019; Shine et al., 2016), sub-states of higher integration or segregation can be identified over time from the connectivity between regions, by establishing a "cartographic profile" based on the graph-theoretical module assignments of each ROI (node). In graph theory, a graph G=(N,K), is a mathematical representation of a network of N nodes connected by K edges. Here, nodes were given by the regions of interest of our parcellation, and edges were given by their functional connectivity. Under these conditions, modules are defined as groups of nodes that are positively correlated with each other, but negatively correlated with nodes belonging to different modules (Sporns and Betzel, 2016).

**Fig. 1:**
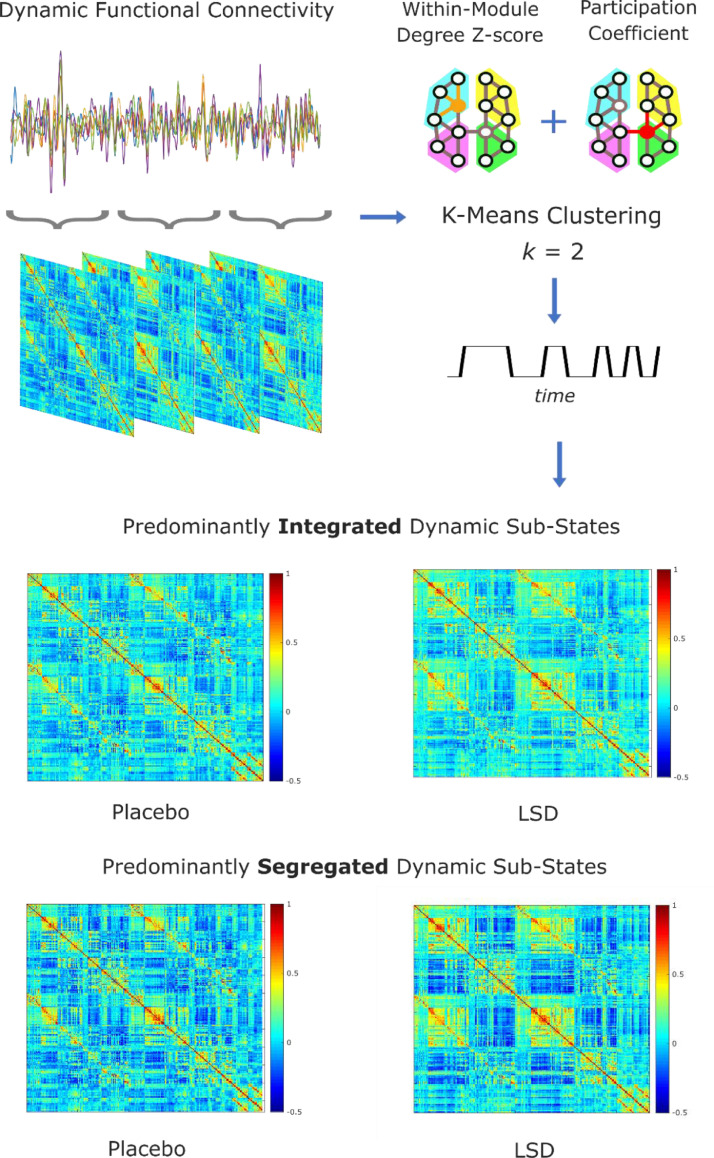
**Derivation of predominantly integrated and segregated sub-states of dynamic functional connectivity.** After obtaining sliding-windows of dynamic functional connectivity, the joint histogram of participation coefficient and within-module degree Z-score is used for *k*-means clustering (with *k =* 2; note that k=2 was also the best clustering solution based on the silhouette criterion for quality of clustering). The cluster with higher (lower) average participation coefficient is then identified as the predominantly integrated (segregated) dynamic sub-state, separately for the Placebo and LSD conditions.

Graph theory was thus applied to the dynamic FC matrices for each participant, to identify modules and subsequently derive the participation coefficient and Z-score of the within-module degree of each ROI, to determine the pattern of connectivity of each node with other modules and with its own module, respectively (Supplementary Materials and Methods). Following previous work (Luppi et al., 2019; Shine et al., 2016), based on the combination of these properties as described by their joint histogram, a k-means clustering algorithm assigned each matrix to one of two clusters (Shine et al., 2016). The clusters with higher and lower average participation coefficient were labelled as the "predominantly integrated" and "predominantly segregated" dynamic sub-states, respectively (Shine et al., 2016) (Fig. 1).

It is important to note at this point that it is still possible and meaningful to quantify the level of segregation in the primarily integrated sub-state, and the integration of the primarily segregated sub-state: these quantities are neither binary, nor mutually exclusive (indeed, their balance is considered an important property in networks (Latora and Marchiori, 2001)). A useful analogy would be with temperatures: even after dividing days in "hot" and "cold", one can still sensibly ask whether a yearly increase in average temperature is due to the hot days being hotter, or the cold days being less cold, or a uniform increase in the temperature of both.

For each subject, sub-state-specific FC centroid matrices were computed, as the element-wise median of the dynamic FC matrices assigned to each sub-state. The proportion of time spent in each sub-state was also quantified, as the number of timepoints assigned to that cluster, over the total number of timepoints.

Finally, the entropy of the pattern of predominantly integrated and segregated sub-states was measured using a naive estimator, which computes probability *p(x)* as the frequency of occurrence of each *x* in the sample, and computes entropy as

(1) $$H\;=\;-\;\sum_{x}p\left(x\right)\;\log_{2}p\left(x\right)$$

### 2.6 Validation of dynamics against stationary null model

Using the cartographic profile method employed here, (Shine et al., 2016) previously demonstrated that the resting brain fluctuates more frequently than a stationary null model, indicating the presence of genuine dynamics in the data. Nevertheless, here we also validated this observation in our own data, using a Vector Autoregressive (VAR) model to generate surrogate timeseries which are stationary by construction. Since fitting a single multi-dimensional VAR that simultaneously considered the covariance between all pairs of regional timeseries was computationally infeasible, we followed (Zalesky et al., 2014), fitting two-dimensional VAR models separately for each pair of ROIs of each participant in each condition. The VAR model order was set to 4, which was chosen to correspond to the same temporal lag of approximately 8s (given our TR of 2s) used by (Shine et al., 2016) and (Zalesky et al., 2014). Sliding-windows dynamic functional connectivity followed by the cartographic profile was then applied to the surrogate data of each participant and condition, and the proportion of time spent in the predominantly integrated sub-state was compared between the empirical data and the stationary surrogates.

### 2.7 Characterisation of dynamic functional brain networks

Previous research has shown that several properties of dynamic functional brain networks are sensitive to pharmacological perturbations of consciousness (Barttfeld et al., 2015; Demertzi et al., 2019; Hutchison et al., 2014; Lord et al., 2019; Luppi et al., 2019). However, it is currently not known whether the effects of LSD on functional brain networks are distributed equally between primarily integrated and segregated sub-states, or whether one or the other is especially susceptible to the effects of this serotonergic psychedelic. Therefore, we set out to investigate whether integrated and segregated sub-states of dynamic functional connectivity are also involved in the altered state of consciousness induced by LSD.

### 2.8 Measures of interest

#### 2.8.1 Prevalence of Anticorrelations

Both anaesthesia and disorders of consciousness are known to suppress anticorrelations between brain regions (Amico et al., 2017; Boveroux et al., 2010; Di Perri et al., 2018, 2016; Guldenmund et al., 2013; Luppi et al., 2019); this phenomenon has also been observed with various psychedelics, including LSD (Carhart-Harris et al., 2016, 2012; Roseman et al., 2014; Tagliazucchi et al., 2016b, 2014), apparently contradicting the claim that the psychedelic state should occupy the opposite end of a continuum as loss of consciousness (Carhart-Harris et al., 2014). However, recent evidence indicates that reduced anticorrelations during loss of consciousness are not uniform in time: rather, they are primarily observed during predominantly integrated dynamic sub-states (Luppi et al., 2019). To determine whether this is also the case for the psychedelic state induced by LSD, we therefore measured the prevalence of anticorrelations in each dynamic sub-state, as the proportion of negative edges, out of the total number of edges in the functional connectivity matrix (excluding the diagonal), separately for the integrated and segregated states.

#### 2.8.2 Structural-functional similarity

Previous results from dynamic functional connectivity have shown that the state of suppressed consciousness induced by anaesthetics, such as the GABA-ergic agent propofol, corresponds to increased similarity between certain sub-states of dynamic functional connectivity and the underlying pattern of anatomical connections (Barttfeld et al., 2015). In order to evaluate the similarity of integrated and segregated states of functional connectivity to the underlying structural connectivity, we computed the correlation between each subject's FC matrix corresponding to the primarily integrated or segregated dynamic sub-state, on the one hand, and the matrix of structural connectivity obtained from a group-average template of 1021 subjects from the Human Connectome Project (Supplementary Materials and Methods), parcellated according to the same parcellation as the functional data. Each matrix of functional connectivity was thresholded proportionally using the same density as the structural connectivity matrix, to ensure that both matrices would have the same number of non-zero entries. Then, the upper triangular portion of each connectivity matrix (structural and functional) was flattened into a vector, and the Spearman correlation coefficient between these two vectors was computed, as our measure of similarity between functional and structural connectivity.

As an alternative method to estimate the similarity between structural and functional patterns of connectivity, we also computed the Hamming distance between the binarized connectivity patterns of each ROI in the functional and anatomical connectivity matrices (Tagliazucchi et al., 2016a). The Hamming distance between vectors *a* and *b* is computed as the number of symbol substitutions required to turn one binary vector into another (normalised by their length). In the present application, it measures the proportion of connections that need to be changed before the two connectivity patterns become the same: thus, whereas correlation measures similarity, the Hamming distance measures dissimilarity. This analysis was performed for each ROI in the parcellation; by averaging over all ROI values, we obtained a value of structural-functional connectivity distance. Both correlation and Hamming distance analysis were performed separately for the matrices of integrated and segregated sub-states.

#### 2.8.3 Small-world propensity

A small-world network combines the presence of tightly interconnected clusters (characterising lattice networks, and theorised to support specialised processing) with a short characteristic path length (a key feature of random networks, facilitating integration between different clusters). Thus, small-worldness represents a mark of optimal balance between global and local processing. Small-worldness of dynamic sub-states has been shown to decrease during anaesthesia and in patients with disorders of consciousness, specifically during the integrated sub-state (Luppi et al., 2019). Therefore, we hypothesised that the opposite may occur following LSD administration.

We adopted the measure of small-world propensity recently developed by (Muldoon et al., 2016), which provides a theoretically principled way to quantify and compare the extent that different networks exhibit small-world structure, while accounting for network density. The small-world propensity, φ, is designed to quantify the extent that a network displays small-world organisation by taking into account the deviation of the network's empirically observed clustering coefficient, *C_obs_*, and characteristic path length, *L_obs_*, from equivalent lattice (*C_latt_, L_latt_*) and random (*C_rand_, L_rand_*) networks.

(2) $$\varphi \;=\;1\;-\;\sqrt{\frac{\Delta_{C}^{2}\;+\;\Delta_{L}^{2}}{2}}$$

where

(3) $$\Delta_{C}=\frac{C_{latt}\;-\;C_{obs}}{C_{latt}\;-\;C_{rand}}$$

(4) $$\Delta_{L}=\frac{L_{obs}\;-\;L_{rand}}{L_{latt}\;-\;L_{rand}}$$

Thus, $\Delta_{C}$ and $\Delta_{L}$ quantify the fractional deviation of the empirically observed clustering coefficient and characteristic path length, from the corresponding null models according to the definition of a small-world network: namely, a lattice network for the clustering coefficient, and a random network for the characteristic path length (Muldoon et al., 2016) (see Supplementary Materials and Methods for the mathematical details of these graph-theoretical measures).

Following (Muldoon et al., 2016), we further bound both measures of fractional deviation between 0 and 1 (to account for the possibility of empirical networks exceeding the corresponding null models), by setting negative values of $\Delta_{C}$ or $\Delta_{L}$ to 0, and values that exceed unity to be exactly 1. In turn, this ensures that the resulting values of small-world propensity will also be bounded between 0 and 1.

Small-world propensity is then interpreted as follows: both a large $\Delta_{C}$ or $\Delta_{L}$ would indicate large deviation of the network's properties from the corresponding properties that define small-world organisation. Thus, large $\Delta_{C}$ or $\Delta_{L}$ would lead to the measure of small-world propensity becoming closer to zero. Conversely, if a network exhibits both the high clustering coefficient of a lattice, and the low path length of a random network (thereby satisfying both requirements of the small-world network definition), then it will have low $\Delta_{C}$ and low $\Delta_{L}$, and the small-world propensity as a whole will be closer to 1. Hence, higher small-world propensity intuitively indicates better adherence to the requirements of a small-world network.

Unlike the small-world index of (Humphries and Gurney, 2008), small-world propensity is not intended as a way to determine, in absolute terms, whether or not a network exhibits small-world structure. Rather, this metric is better suited to compare on a continuous scale the degree of small-world organisation exhibited by different networks (Muldoon et al., 2016).

#### 2.8.4 Functional network construction

Anatomical connectivity in the human brain is known to be sparse (Sporns, 2011), and weak edges may represent false positives due to measurement error, which may obscure the network's true topology (Rubinov and Sporns, 2010). Thus, we constructed brain networks by thresholding the corresponding functional connectivity matrices so that only a fixed proportion of the strongest (positive) connections were retained, setting all above-threshold edge weights to unity, and the rest to zero. By enforcing the same number of edges across participants and conditions, and assigning unit weight to each edge, this construction procedure ensures that any observed differences will be due solely to the networks’ topology (i.e. which network elements are connected).

Since there is currently no gold standard for the threshold level to use, to ensure that our results would not be dependent on the specific threshold chosen we thresholded each FC matrix at density levels ranging between 10% and 25%, sampled in steps of 5%. Such density levels are commonly employed in graph-theoretical analysis of functional brain networks (Cohen and D'Esposito, 2016), since they tend to produce plausibly sparse, cost-efficient networks (Achard and Bullmore, 2007). To ensure robustness of the results, values of small-world propensity for thresholded networks were averaged across thresholds for each subject and condition before analysis.

However, edge weight may be expected to carry biological meaning, as the extent of communication between different regions. Since the measure of small-world propensity adopted here can be applied on weighted networks, we also repeated our analysis including all non-negative edges (negative edges were set to zero because this measure depends on calculations of shortest paths between nodes, and therefore requires graphs with strictly positive edges (Rubinov and Sporns, 2011, 2010)).

#### 2.8.5 Network functional complexity

To quantify the functional diversity of functional brain networks, we also applied the recently introduced measure of “Functional Complexity” (Zamora-López et al., 2016). Following (Zamora-López et al., 2016), we compute the functional complexity C of the network as the difference between the observed distribution *p(r_ij_)* and the uniform distribution. If *p(r_ij_)* is estimated in *m* bins, the uniform distribution is

(5) $${\bar{p}}_{\mu }=\;\frac{1}{m}$$

for all bins *μ* = 1, 2, …, *m*. Hence, functional complexity is quantified as the sum of the differences of the two distributions over the bins:

(6) $$C\;=\;1\;-\;\frac{1}{C_{m}}\sum_{\mu =1}^{m}\left|p_{\mu }\left(r_{ij}\right)\;-\;\frac{1}{m}\right|$$

where |•| represents the absolute value operator and $C_{m}=2\frac{m-1}{m}$ is a normalisation factor that represents the extreme case in which the *p(r_ij_)* is a Dirac-delta function δ_m_. That is, when all *r_ij_* values fall in the same bin, which can occur either because the nodes are mutually independent, or globally synchronised.

Functional complexity was computed for each subject in each condition, separately for the integrated and segregated state. Since this analysis considers connectivity as the extent of coupling between distinct elements of the system, negative values were excluded, as well as removing the diagonal entries.

### 2.9 Statistical analysis

All statistical tests were two-tailed. Correlations between brain measures and subjective ratings were computed with Spearmsn's rank-based correlation coefficient, ρ. The statistical significance of within-subjects differences between the placebo and LSD conditions was tested using a robust linear model, implemented in the MATLAB function *fitlm*.

Since head motion (mean framewise displacement) was significantly higher in the LSD than placebo condition (Placebo mean = 0.079, SD = 0.026; LSD mean = 0.129, SD = 0.039; *t*(14) = -6.34, *p* < 0.001; repeated-measures t-test; Supplementary Figure 1), in addition to our rigorous denoising procedure we also included mean framewise displacement as a covariate of no interest in our statistical analyses, to further account for the potential confounding effects of head motion.

#### 2.9.1 Network based statistic

The network-based statistic (NBS) approach (Zalesky et al., 2010) was used to investigate the statistical significance of LSD-induced alterations on the functional brain networks, for time-averaged functional connectivity and for the predominantly integrated and segregated sub-states. This nonparametric statistical method is designed to control the family-wise error due to multiple comparisons, for application to graph data. Connected components of the graph are identified from edges that survive an a-priori statistical threshold (F-test; here we set the threshold to an intensity value of 10). In turn, the statistical significance of such connected components is estimated by comparing their topological extension against a null distribution of the size of connected components obtained from non-parametric permutation testing. This approach rejects the null hypothesis on a component-basis, and therefore achieves superior power compared to mass-univariate approaches (Zalesky et al., 2010). Once again, mean framewise displacement was included as a covariate of no interest to further mitigate its potential confounding effects on our analyses. To ensure the robustness of our results to the choice of the threshold parameter, we also replicated them with an intensity-based F-threshold of 12, and with an extent-based F-threshold of 10.

#### 2.9.2 Correlations with subjective experiences

Correlations between brain measures and subjective experiences (ASC scale as well as VAS) were obtained using Spearman's non-parametric rank-based correlation coefficient ρ, implemented in the R package *ggstatsplot*. In addition to subjective ratings, we also correlated each brain measure with the mean framewise displacement, to investigate the possible effects of motion on our results. Due to the exploratory nature of this analysis, we did not perform correction for multiple comparisons, thus considering an alpha level of 0.05 for statistical significance. Nevertheless, we accounted for potential false positive results by only interpreting correlations that were significant across all three parcellations, and were therefore robust to both type and size of parcellation.

## 3 Results

### 3.1 Preserved temporal balance of integration and segregation under LSD

**Fig. 2:**
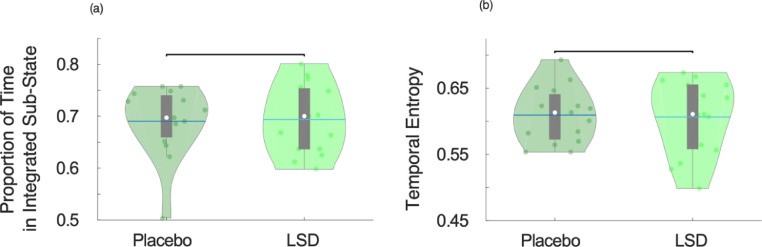
**Temporal properties of brain sub-states under placebo and LSD.** (a) Violin plots of the proportion of total time spent in the predominantly integrated sub-state. (b) Violin plots of the sample entropy of the alternation of integrated and segregated sub-states, over the entire scan duration. Violin plots indicate the distribution of participants in each condition (coloured circles). White circle, mean; blue center line, median; box limits, upper and lower quartiles; whiskers, 1.5x interquartile range.

Studying alterations in brain function induced by LSD offers a way to relate its potent psychedelic effects to their underlying neurobiological correlates. In particular, the focus of this study was on the effects of LSD on the dynamics of two fundamental properties of the brain: integration and segregation. Thus, we employed an a priori clustering of dynamic functional connectivity into two sub-states, for both LSD and placebo (Fig. 1). Although group-level differences between the two states were subtle (especially for the placebo condition), multiple control analyses reassured us that our cartographic profile approach was capturing genuinely distinct dynamical states. First, the silhouette criterion for quality of clustering (measured using MATLAB's *evalclusters* command) selected *k* = 2 as the most appropriate clustering of dynamic functional connectivity across values of *k* ranging between 2 and 7 (Supplementary Figure 2), for each subject and condition, thus providing data-driven support for our hypothesis-driven clustering of dynamic FC into two sub-states (predominantly integrated and segregated). Second, the proportion of time spent in the globally integrated sub-state during the placebo condition (*M* = 0.69, *SD* = 0.07) was consistent with the data on healthy volunteers reported by Shine and colleagues (Shine et al., 2016), and by (Luppi et al., 2019), providing an additional sanity check on the key method underlying the present results (Fig. 2a and Table 1) (note that the proportion of time spent in the primarily segregated sub-state is just the complement of the proportion of time spent in the integrated sub-state, since each timepoint belongs to either one or the other sub-state). This proportion was also preserved under the effects of LSD (Fig. 2a and Table 1) suggesting that the effects of LSD do not manifest as altered temporal structure of brain sub-states over time.

**Table 1:** Statistical estimates of the comparison between LSD and placebo, for time-averaged FC and for the predominantly integrated and segregated dynamic sub-states, for the Schaefer-232 parcellation

Measure	Estimate	SE	tStat	pValue	Sig
**Time-Averaged Functional Connectivity**					
**Functional Complexity**	0.040	0.017	2.418	0.023	*
**Proportion of Anticorrelations**	-0.061	0.027	-2.259	0.032	*
**Structural-Functional Correlation**	-0.008	0.007	-1.222	0.232	-
**Average Structural-Functional Hamming Distance**	0.001	0.001	1.200	0.241	-
**Small-World Propensity - Binary**	0.018	0.008	2.318	0.028	*
**ΔC - Binary**	-0.046	0.015	-3.011	0.006	**
**ΔL – Binary**	0.048	0.012	4.112	0.000	***
**Small-World Propensity - Weighted**	0.058	0.016	3.604	0.001	**
**ΔC - Weighted**	-0.129	0.037	-3.448	0.002	**
**ΔL – Weighted**	0.045	0.013	3.536	0.001	**
**Predominantly Integrated Dynamic Sub-State**					
**Functional Complexity**	0.037	0.020	1.795	0.084	-
**Proportion of Anticorrelations**	-0.057	0.031	-1.812	0.081	-
**Structural-Functional Correlation**	-0.008	0.008	-0.934	0.359	-
**Average Structural-Functional Hamming Distance**	0.001	0.001	0.904	0.374	-
**Small-World Propensity - Binary**	0.013	0.009	1.426	0.165	-
**ΔC - Binary**	-0.026	0.016	-1.688	0.103	-
**ΔL – Binary**	0.027	0.010	2.691	0.012	*
**Small-World Propensity - Weighted**	0.046	0.021	2.146	0.041	*
**ΔC - Weighted**	-0.088	0.042	-2.104	0.045	*
**ΔL – Weighted**	0.032	0.013	2.455	0.021	*
**Predominantly Segregated Dynamic Sub-State**					
**Functional Complexity**	0.042	0.019	2.221	0.035	*
**Proportion of Anticorrelations**	-0.044	0.017	-2.567	0.016	*
**Structural-Functional Correlation**	-0.019	0.008	-2.456	0.021	*
**Average Structural-Functional Hamming Distance**	0.003	0.001	2.467	0.020	*
**Small-World Propensity - Binary**	0.024	0.010	2.481	0.020	*
**ΔC - Binary**	-0.067	0.021	-3.158	0.004	**
**ΔL – Binary**	0.069	0.019	3.606	0.001	**
**Small-World Propensity - Weighted**	0.062	0.018	3.542	0.001	**
**ΔC - Weighted**	-0.156	0.042	-3.716	0.001	***
**ΔL – Weighted**	0.064	0.017	3.861	0.001	***
**Integration-Segregation Dynamics**					
**Integration-Segregation Temporal Entropy**	0.023	0.024	0.984	0.334	-
**Proportion of Time in Integrated State**	-0.032	0.030	-1.071	0.294	-

Estimate, estimated difference between conditions; SE, standard error; pValue, p-value; tStat, test statistic; * *p* < 0.05; ** *p* < 0.01 *** *p* < 0.001.

Third, in the placebo condition the proportion of time spent in the integrated sub-state was significantly greater for empirical data (*M* = 0.69, *SD* = 0.07) than stationary data generated by a VAR model for each participant (*M* = 0.51, *SD* = 0.25, *t*(14) = 2.42, *p* = 0.030; repeated-measures t-tests), which was not different from random (i.e. 50%) (*t*(14) = 0.22, *p* = 0.826). Likewise, under the effects of LSD the proportion of time spent in the integrated sub-state was also significantly greater for empirical data (*M* = 0.69, *SD* = 0.07) than stationary data generated by a VAR model for each participant (*M* = 0.55, *SD* = 0.20, *t*(14) = 2.91, *p* = 0.014; repeated-measures t-tests), which again was not different from random (i.e. 50%) (*t*(14) = 1.00, *p* = 0.332). (Supplementary Figure 3). Thus, for both placebo and LSD, the proportion of time spent in the predominantly integrated sub-state was consistent with previous observations in awake, healthy individuals (Luppi et al., 2019; Shine et al., 2016) and reliably greater than for corresponding stationary null models, indicating the presence of dynamics in the functional connectivity – in line with previous results employing the same cartographic profile method (Shine et al., 2016).

Since LSD and other psychedelics are known to increase the entropy of certain dimensions of brain function (Lebedev et al., 2016; Tagliazucchi et al., 2014; Viol et al., 2017), we investigated whether the entropy of the temporal alternation of the two sub-states (primarily integrated and segregated) over the entire scan duration would also exhibit this phenomenon under LSD. However, the sequence of alternations between the two sub-states did not exhibit significant changes in its temporal entropy under the effects of LSD compared with placebo (Fig. 2b and Table 1).

Although the temporal structure of integrated and segregated sub-states was not affected by LSD, prior research suggests that altered states of consciousness have differential effects on functional brain networks during the integrated and segregated sub-states (Luppi et al., 2019). Thus, we sought to determine whether the effects of LSD on the brain can only be seen when considering the entire duration of the scanning period (i.e. when considering so-called ‘time-averaged’ FC), or whether different effects of LSD on brain function are manifested during dynamic sub-states of high or low integration or segregation.

We therefore investigated how each of these two sub-states was affected by LSD, in terms of its functional connectivity, as well as its network properties, given their proposed relevance for consciousness (Dehaene et al., 2011). For purposes of comparison with more traditional analyses, we also carried out these analyses for time-averaged functional connectivity, spanning the entire concatenated scan duration.

### 3.2 Time-specific effects of LSD on brain connectivity

**Fig. 3:**
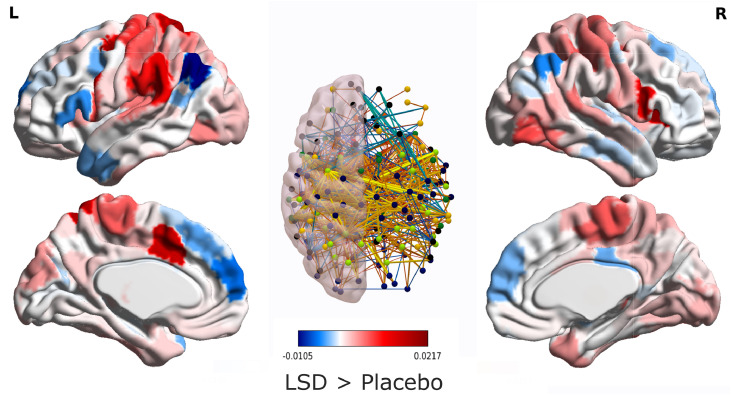
**LSD-induced reorganisation of functional connectivity in the predominantly segregated dynamic sub-state.** Center: brain networks of significant functional connectivity differences (*r* values) between placebo and LSD, obtained from the Network Based Statistic with an intensity-based F-threshold of 10; Red-yellow indicates LSD > placebo; blue-green indicates LSD < placebo. Sides: surface projection of the total change in connectivity (sum of significant connection changes) for each region of the augmented Schaefer-232 atlas.

Network alterations induced by LSD can be identified by determining whether specific pairs of brain regions exhibit changes in the strength of their functional connectivity. Network-level connectivity alterations under LSD (as indicated by the Network Based Statistic) were evident both from time-averaged FC, and also in the predominantly segregated sub-state of dynamic functional connectivity, respectively (Fig. 3 and Supplementary Figure 4). For time-averaged FC, a predominance of global FC increases was observed, as well as decoupling between prefrontal and posterior and temporal regions, as previously observed with psilocybin (Roseman et al., 2014). Crucially, our temporal analysis allowed us to determine that FC alterations induced by LSD are sub-state-dependent: no significant alterations in functional connectivity were observed during the predominantly integrated dynamic sub-state. In contrast, the predominantly segregated dynamic sub-state displayed both increases as well as decreases in functional connectivity (Fig. 3). Intriguingly, although some of these FC changes were also observed in time-averaged FC (e.g. disconnections between visual regions), others were specific to the segregated sub-state – including most of the disconnections from prefrontal cortex. In particular, the most striking difference was that functional connectivity during the predominantly segregated sub-state decreased overall in the anterior medial prefrontal cortex, which was not observed for time-averaged functional connectivity (which instead displayed stronger reductions in medial orbitofrontal cortex) (Supplementary Figures 5-6).

Overall, the predominantly segregated sub-state was characterised by a net increase in functional connectivity of somatomotor and auditory cortices, and a net decrease in FC of key nodes of the default mode network (medial prefrontal cortex, bilateral angular gyri and temporal poles), in line with previous results from time-averaged FC (Carhart-Harris et al., 2016, 2012). During the segregated sub-state, another key hub of the DMN, the posterior cingulate, reduced its connectivity with visual regions, while increasing it with typically anticorrelated regions from the executive control network. The thalamus also displayed several increases in functional connectivity, with both cortical (orbitofrontal and temporal cortices) and subcortical structures (bilateral amygdalae). These results were robust to the choice and type of threshold chosen for the Network Based Statistic (Supplementary Figures 5-6). They were also robust to the choice of type and size of parcellation, as attested by replicating them using the Brainnetome 246-ROI multimodal parcellation, and the augmented Schaefer-454 parcellation (Supplementary Figures 7-9) – with the main difference being that the NBS reported no significant differences for time-averaged FC when using the Brainnetome atlas (although the same pattern of increases and decreases was observed for the predominantly segregated sub-state).

### 3.3 Structural-functional untethering and reduced anticorrelations in the segregated sub-state

**Fig. 4:**
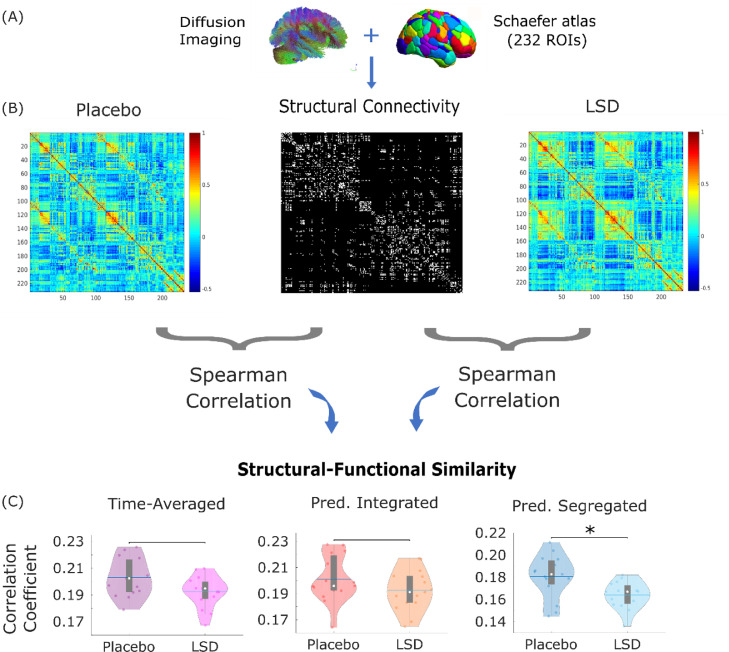
**Structural similarity of time-averaged and dynamic functional brain networks.** (A) Structural connectivity was obtained from the group-averaged DTI of 1021 healthy subjects from the Human Connectome Project, parcellated according to the augmented Schaefer-232 atlas; edges corresponded to the number of streamlines connecting each pair of ROIs. (B) Structural-functional similarity was calculated as the Spearman correlation between vectorised structural and functional connectivity matrices. (C) Comparison of structural-functional correlation between the placebo and LSD conditions, for time-averaged functional connectivity and also for the predominantly integrated and segregated dynamic sub-states. Violin plots indicate the distribution of participants in each condition (coloured circles). White circle, mean; blue center line, median; box limits, upper and lower quartiles; whiskers, 1.5x interquartile range. * *p* < 0.05; * *p* < 0.01;

In addition to changes in the strength of specific network edges (i.e., connections between pairs of brain regions), it can also be advantageous to interrogate properties of the network as a whole, to obtain a more thorough understanding of how alterations in one's state of consciousness arise from perturbations of the brain's network organisation. Previous results have shown that the state of suppressed consciousness induced by anaesthetics, such as the GABA-ergic agent propofol, corresponds to increased similarity between functional connectivity and the underlying pattern of anatomical connections (Tagliazucchi et al., 2016a); however, further evidence from dynamic functional connectivity indicates that this phenomenon unfolds dynamically over time (Barttfeld et al., 2015). We therefore set out to determine whether the structural-functional relationship would also be influenced by LSD in a time-varying fashion (Fig. 4A,B), and may represent a general marker of state of consciousness.

Our results indicated no significant change in the similarity between time-averaged functional connectivity and the underlying structural connectome (Fig. 4C and Table 1). However, dynamic analysis revealed that although LSD did not significantly affect the functional-structural similarity during the primarily integrated sub-state, functional connectivity during the primarily segregated sub-state was significantly less similar to structural connectivity, following LSD administration (Fig. 4C). Thus, there are moments in time (characterised by especially high network segregation) during which LSD induces the opposite effect to anaesthesia, allowing FC to diverge more freely from anatomical constraints. The same results were also observed when using the Hamming distance instead of correlation to quantify structural-functional (dis)similarity: average Hamming distance between function and structure increased under the effects of LSD, corresponding to higher dissimilarity, but only for the segregated sub-state (Supplementary Figure 10). It is also worth noting that for both placebo and LSD, the highest similarity to structural connectivity was observed during the primarily integrated sub-state, in line with the results of Fukushima and colleagues (Fukushima et al., 2018).

Another robust marker of loss of consciousness induced by anaesthesia and disorders of consciousness is the suppression of anticorrelations between brain regions (Amico et al., 2017; Boveroux et al., 2010; Di Perri et al., 2018, 2016; Guldenmund et al., 2013; Luppi et al., 2019); intriguingly, recent evidence indicates that this phenomenon is not uniform over time: rather, it is mainly observed during the predominantly integrated dynamic sub-state. Since reduced anticorrelations have also been observed with various psychedelics, including LSD (Carhart-Harris et al., 2016, 2012; Roseman et al., 2014; Tagliazucchi et al., 2016b, 2014), we next sought to determine whether this phenomenon also differs across dynamic sub-states of functional connectivity.

**Fig. 5:**
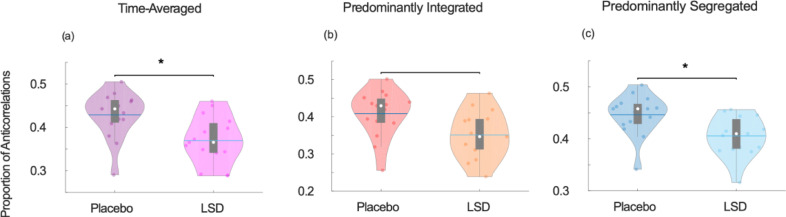
**LSD-induced reduction in the proportion of functional anticorrelations.** (a) Time-averaged functional connectivity. (b) Predominantly integrated dynamic sub-state. (c) Predominantly segregated dynamic sub-state. Violin plots indicate the distribution of participants in each condition (coloured circles). White circle, mean; blue center line, median; box limits, upper and lower quartiles; whiskers, 1.5x interquartile range. * *p* < 0.05.

In line with previous results, we find that LSD reduces the proportion of negative edges across the brain, when considering FC over the full scanning length (Fig. 5a). However, dynamic analysis revealed that once again, this effect is sub-state specific: no effect of LSD on the prevalence of negative edges was observed during the primarily integrated sub-state (Fig. 5b), whereas a significant reduction was observed during the primarily segregated sub-state – the opposite of what was found with loss of consciousness (Luppi et al., 2019) (Fig. 5c) (Table 1).

### 3.4 Increased functional network small-worldness under LSD

When investigating information-processing networks, such as the human brain, it is advantageous to consider what kind of network structure would facilitate the exchange of information. Previous evidence indicates that small-world organisation, a marker of optimal balance between local and global processing (Bassett and Bullmore, 2017), is compromised in a dynamic fashion during loss of consciousness (Barttfeld et al., 2015; Luppi et al., 2019). Therefore, we expected LSD to have the opposite effect on human brain networks.

**Fig. 6:**
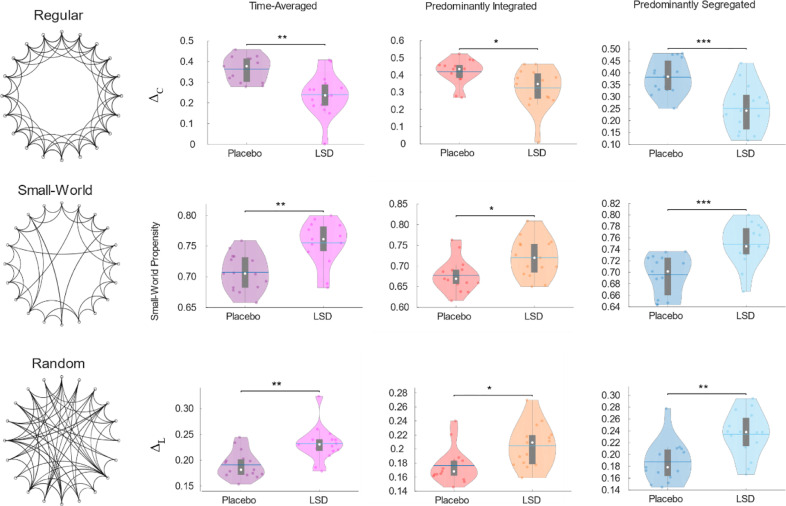
**Increased small-world propensity of time-averaged and dynamic weighted brain networks under LSD.** Violin plots showing the comparison of deviation from a regular (lattice) network in terms of clustering (Δ_C_; top row), small-world propensity (middle row), and deviation from a random network in terms of characteristic path length (Δ_L_), for weighted networks obtained from time-averaged functional connectivity, the predominantly integrated sub-state, and the predominantly segregated dynamic sub-state. White circle, mean; blue center line, median; box limits, upper and lower quartiles; whiskers, 1.5x interquartile range. * *p* < 0.05; ** *p* < 0.01; *** *p* < 0.001.

In line with our hypothesis of opposite effects of LSD compared with loss of consciousness, our results show that LSD induces an increase in the small-world propensity of time-averaged functional brain networks. The increase was only observed during the primarily segregated sub-state (as well as for time-averaged FC) when considering binary networks comprising only the strongest connections (Supplementary Figure 11 and Table 1). However, when the weight of connections between brain regions was taken into account, and weaker links were included, increased small-world propensity under LSD could be detected during both the predominantly segregated and predominantly integrated sub-states of dynamic functional connectivity (Fig. 6 and Table 1).

In both cases, the overall increase in small-world propensity resulted from the balance of two opposing trends: the deviation of characteristic path length from a corresponding random network (Δ_L_) increased under LSD, reducing the network's small-world propensity; however, this effect was more than compensated by a decrease in the deviation of the network's clustering coefficient from that of a regular network (Δ_C_) – resulting in overall increased small-world propensity. In other words, under the effects of LSD, the brain's characteristic path length becomes less similar to that of a random network, but the clustering coefficient becoming more similar to that of a lattice network – recapitulating results obtained in time-averaged networks after ingestion of ayahuasca (Viol et al., 2017).

### 3.5 Increased functional complexity in the segregated sub-state

Finally, recent research has converged in indicating that aspects of spatial and temporal complexity of the brain are enhanced during the psychedelic state induced by LSD, and decreased when consciousness is lost due to anaesthesia or severe brain injury. Thus, brain complexity may provide a proficuous avenue for grounding the mind-altering effects of LSD according to the underlying neurobiology. Therefore, we investigated whether the well-known effects of LSD on brain complexity are uniform in time, or depend on the brain's sub-states of high integration or segregation, and how this is related to alterations observed from time-averaged FC results. To do this, we adopted a recently developed measure of functional complexity, allowing us to quantify the complexity of the pattern of whole-brain connectivity during each dynamic sub-state (Zamora-López et al., 2016). Being predicated in space rather than time, this measure provides a complementary quantification of complexity in the brain to our previous measure of entropy, which assessed the unpredictability of the alternation between dynamic sub-states over time.

**Fig. 7:**
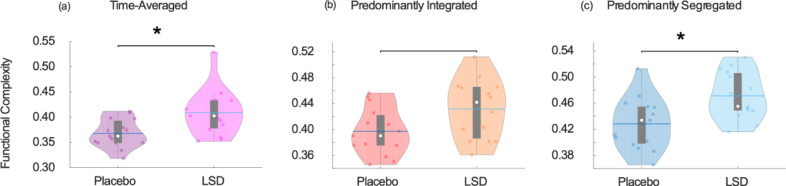
**Increased functional complexity under LSD.** (a) Comparison of functional complexity for time-averaged functional connectivity. (b) Comparison of functional complexity for the predominantly integrated dynamic sub-state. (c) Comparison of functional complexity for the predominantly segregated dynamic sub-state. Violin plots show the distribution of H-index values for each group. White circle, mean; blue center line, median; box limits, upper and lower quartiles; whiskers, 1.5x interquartile range. * p < 0.05.

Our results indicate overall increased functional complexity during the psychedelic state induced by LSD (Fig. 7a and Table 1). However, dynamic analysis once again revealed that this effect was not distributed uniformly in time: it was not observed during the primarily integrated sub-state (Fig. 7b), instead only manifesting itself during the primarily segregated sub-state (Fig. 7c).

### 3.6 Robustness to parcellation type and size

To ensure the robustness of our results pertaining to network properties, we replicated them using two alternative ways of defining brain network nodes (Luppi and Stamatakis, 2020), as already done for the NBS results. Specifically, to ensure robustness to the size of parcellation, we employed a finer-grained version of the multi-scale Schaefer functional cortical atlas (Schaefer et al., 2018) (with 400 cortical ROIs instead of 200) supplemented with a corresponding finer-grained version of the Melbourne functional subcortical atlas (Tian et al., 2020) (with 54 subcortical ROIs instead of 32). Conversely, to ensure robustness to the type of parcellation, we employed the Brainnetome atlas (Fan et al., 2016), which comprises 210 cortical and 36 subcortical ROIs (similar in number to the augmented Schaefer-232), obtained from anatomical and functional connectivity (Supplementary Tables 2-3).

The same patterns of results were obtained using these different parcellations as with the Schaefer-232, with the following exceptions: for time-averaged FC, significantly increased difference from structural connectivity (lower correlation and higher average Hamming distance) was observed with the Schaefer-454 parcellation. For the predominantly integrated sub-state, the Brainnetome-246 parcellation narrowly failed to detect statistically significant differences in Δ_C_ and Δ_L_ of weighted networks – even though their combination was still reflected in a significant increase of small-world propensity.

### 3.7 Correlations with subjective experiences

Our exploratory analysis of the correlations between LSD-induced changes in brain measures and subjective ratings revealed several significant correlations (Supplementary Figure 12). However, the majority of significant correlations were only observed with a single parcellation. Therefore, we have chosen to only discuss correlations that were reproducible (in terms of providing consistent significant results) across all three parcellations (correlation scores and associated *p*-values are reported for brain measures obtained from the Schaefer-232 atlas).

Using this criterion, no significant correlations were found between subjective ratings and our brain measures of interest, when considering FC from the full scanning duration (Supplementary Figure 12). However, dynamic analysis revealed that consistent significant correlations were obtained for both the predominantly integrated and segregated dynamic sub-states.

**Fig. 8:**
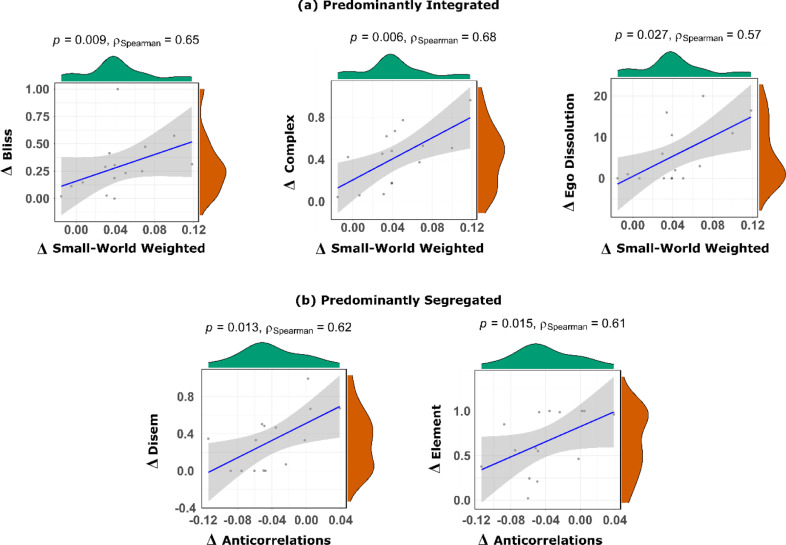
**Significant correlations between LSD-induced changes in subjective ratings and brain measures.** (a) Scatterplots and marginal distributions of the change (LSD – Placebo) in weighted small-world propensity during the predominantly integrated dynamic sub-state, versus the change in subjective scores of blissful feeling, complex imagery and ego dissolution. (b) Scatterplots and marginal distributions of the change (LSD – Placebo) in proportion of anticorrelations during the predominantly segregated dynamic sub-state, versus the change in subjective scores of feeling of disembodiment, and elementary imagery. Brain measures are from data parcellated according to the Schaefer-232 atlas. Correlation scores are Spearman's rho; shaded regions indicate 95% confidence. Bliss: ASC blissful feeling score. Complex: ASC complex imagery score. Ego Dissolution: VAS ego-dissolution score. Disem: ASC feeling of disembodiment score. Element: ASC elementary imagery score.

For the predominantly integrated sub-state, consistent correlations were obtained for the change in weighted small-world propensity, which was significantly positively correlated with ASC subjective ratings of blissful state (Spearman's ρ = 0.65, *p* = 0.009, CI_95%_ [0.20, 0.867, *N =* 15), ASC complex imagery (Spearman's ρ = 0.68, *p* = 0.006, CI_95%_ [0.25, 0.88], *N =* 15) and also the VAS score quantifying the feeling of ego dissolution (Spearman's ρ = 0.57, *p* = 0.027, CI_95%_ [0.08, 0.84], *N =* 15) (Fig. 8a).

Consistent significant correlations were also observed across all three parcellations between the change in prevalence of anticorrelations in the predominantly segregated sub-state, and the ASC disembodiment score (Spearman's ρ = 0.62, *p* = 0.013, CI_95%_ [0.16, 0.86], *N =* 15) and ASC elementary imagery score (Spearman's ρ = 0.61, *p* = 0.015, CI_95%_ [0.15, 0.86], *N =* 15) (Fig. 8b).

Finally, we note that none of our brain measures significantly correlated with mean framewise displacement (a key motion parameter), when using the Schaefer parcellations (and only Δ_L_ from binary networks from the predominantly integrated sub-state showed such a correlation, in the Brainnetome parcellation), supporting the adequacy of our rigorous denoising procedures (Supplementary Figure 12).

## 4 Discussion

Due to its potent temporary effects on both the human mind and brain, the serotonergic psychedelic LSD represents a powerful method for connecting psychological phenomena to their neurobiological correlates, and improving our understanding of both. However, by definition, the stream of consciousness is dynamic and always flowing: ignoring this temporal aspect may neglect a key aspect of consciousness and its psychedelic-induced alterations. Focusing on two fundamental properties of both the human mind and brain – integration and segregation - here we combined fMRI dynamic functional connectivity and graph theory to investigate how the classic serotonergic psychedelic LSD modifies brain function and its dynamics under task-free, eyes-closed resting-state conditions, by investigating the network characteristics in a temporally sensitive way.

Highlighting the crucial importance of considering brain dynamics when investigating states of altered consciousness, our first observation was that the consciousness-altering effects of LSD show prominent temporal aspects. In particular, these effects do not influence the overall allocation of time between predominantly integrated and segregated sub-states – echoing the results obtained in both anaesthesia and disorders of consciousness (Luppi et al., 2019). Rather, LSD differentially affects the network properties of predominantly integrated and segregated sub-states.

A robustly observed neural marker of the psychedelic experience induced by various drugs is the suppression of anticorrelations between brain regions (Carhart-Harris et al., 2016, 2012; Roseman et al., 2014; Tagliazucchi et al., 2016b, 2014). Reduced anticorrelations are also reliably observed during loss of consciousness induced by anaesthesia or brain injury (Amico et al., 2017; Boveroux et al., 2010; Di Perri et al., 2018, 2016; Guldenmund et al., 2013; Luppi et al., 2019); however, our findings resolve this apparent paradox. Recent evidence indicates that reduced anticorrelations in anaesthesia and disorders of consciousness are most prominent during the integrated sub-state (Luppi et al., 2019), whereas the present results reveal that LSD suppresses anticorrelations during the predominantly segregated sub-state. Indeed, the correlation of this effect with subjective experiences of disembodiment and elementary imagery was selective for the segregated sub-state. Thus, by considering brain network dynamics we can discover that apparently similar effects of LSD and loss of consciousness (i.e. reduced anticorrelations in time-averaged FC) actually have their origins in different dynamic sub-states – thereby reconciling apparently opposite observations, and demonstrating the value of taking a dynamic approach to the study of altered brain function.

It has been postulated that the functional complexity of the resting human brain operates near the maximum supported by the underlying structural connectome (Zamora-López et al., 2016). Here, we show that the state of altered consciousness induced by LSD corresponds to an additional, abnormal increase in functional complexity of the brain as a result of LSD. Remarkably, this effect is not uniform in time, but rather, it is only observed during moments when the brain is characterised by a predominantly segregated pattern of functional connectivity – coinciding with reduced similarity between functional connectivity and the underlying pattern of structural connections (which is the opposite of what is typically found during anaesthesia (Barttfeld et al., 2015; Lee et al., 2019; Ma et al., 2017; Uhrig et al., 2018)). Further highlighting the importance of considering network dynamics, this structural-functional decoupling is exclusively observed during the predominantly segregated sub-state (i.e. this effect is not apparent when only considering time-averaged FC). At baseline (placebo condition), the predominantly segregated sub-state is already more decoupled from structural connectivity than the integrated sub-state (in line with the results of (Fukushima et al., 2018)), and also exhibits larger functional complexity. Thus, LSD appears to induce especially complex patterns of functional connectivity by inducing additional decoupling of FC from the underlying structural connectome, precisely during those times when structural-functional coupling is already at its lowest. This evidence is also consistent with previous results obtained by Atasoy and colleagues in the same LSD dataset, as well as in psilocybin, by using an alternative method to study brain dynamics, known as connectome harmonic decompositions (CHD) (Atasoy et al., 2018, 2017). This technique decomposes brain activity into a set of progressively more fine-grained patterns derived from the human connectome (termed “connectome harmonics” (Atasoy et al., 2016)); (Atasoy et al., 2018, 2017) observed that LSD and psilocybin induce a reduction in the contribution of coarse-grained harmonics – which according to recent evidence are precisely the harmonics most strongly coupled to the underlying structural connectome (Preti and Van De Ville, 2019). Thus, two different ways of investigating brain dynamics converge to reveal a psychedelic-induced decoupling of function from structure.

Reduced similarity between structural and functional connectivity indicates that under the effects of LSD, brain regions interact functionally in a way that is less constrained than usual by the presence or absence of an underlying anatomical connection. Anatomical brain connectivity may be understood as representing the organism's expectation about which brain regions should be exchanging information with each other, sculpted by the combined effects of evolution and experience, in accordance with Donald Hebb's famous dictum that “neurons that fire together, wire together”. In other words, anatomical connectivity may be seen as representing a prior (in Bayesian terms) on functional connectivity. When considered in this way, the increased correlation between structural and functional connectivity during anaesthetic-induced unconsciousness (Barttfeld et al., 2015; Lee et al., 2019; Ma et al., 2017; Uhrig et al., 2018) would reflect the fact that incoming external information is being processed only to a minimal degree (Ní Mhuircheartaigh et al., 2013) and therefore the patterns of functional connectivity are primarily dictated by structurally-encoded priors. In contrast, the reduced structural-functional correlation that we observed during the psychedelic state induced by LSD would be consistent with a reduced effect of priors on cognition, as postulated by the REBUS framework (Carhart-Harris and Friston, 2019). Being less constrained by pre-existing priors due to the effects of LSD, the brain is free to explore a variety of functional connectivity patterns that go beyond those dictated by anatomy – presumably resulting in the unusual beliefs and experiences reported during the psychedelic state, and reflected by increased functional complexity. Importantly, this observation is only possible thanks to our decomposition of functional connectivity into predominantly integrated and segregated dynamic sub-states.

The large-scale reorganisation of functional connectivity during the segregated sub-state crucially includes LSD-induced disconnections between posterior and frontal regions - especially the left anterior medial prefrontal cortex. This is particularly intriguing because anterior mPFC has been consistently and specifically implicated in the cognitive process known as reality monitoring, which is the ability to correctly discriminate whether information is derived exogenously (from perceptual processes) or generated endogenously (from thoughts and imagination) (Simons et al., 2017). Dysfunction of this process is thought to underlie the auditory and visual hallucinations that are a prevalent feature of schizophrenia (Simons et al., 2017). Anterior mPFC is consistently found to be both hypoactive during tasks involving reality monitoring in patients with schizophrenia, who experience auditory and visual hallucinations (Simons et al., 2017). Intriguingly, in schizophrenic patients, the same region also exhibits disconnections with posterior regions (Mechelli et al., 2007; Wang et al., 2011), akin to what was observed in the present study as a result of LSD administration. Remarkably, just like the aforementioned reduction in structural-functional coupling, mPFC disconnection was also exclusively observed in the predominantly segregated sub-state, and not when considering time-averaged FC (which instead exhibited disconnections from a more ventral portion of PFC). Thus, we speculate that the anterior mPFC disconnections induced by LSD during the predominantly segregated state, may correspond to attenuated engagement of top-down reality monitoring processes. This is again in accordance with what the REBUS/Anarchic Brain hypothesis of psychedelic action (Carhart-Harris and Friston, 2019) postulates, corresponding to a “suspension of disbelief” right at the moment when the most diverse patterns of connectivity are being explored, and when the functional connectome is minimally constrained by the hard-wired connections between brain regions. This hypothesis may be empirically tested in future work, directly assessing reality monitoring processing during the psychedelic state, for instance by means of experience sampling approache (Petitmengin and Lachaux, 2013).

More broadly, these observations are also consistent with the main tenet shared by theories of psychedelic action from the 19^th^, 20^th^ and 21^st^ century: spanning from the entropic brain hypothesis and predictive processing framework (the two main pillars of the REBUS/Anarchic brain hypothesis) but also including psychoanalytic and filtration accounts: namely, that psychedelics “perturb adaptive mechanisms which normally constrain perception, emotion, cognition, and self-reference” (Swanson, 2018), page 16).

Finally, both the predominantly integrated and segregated sub-states exhibited increased small-world propensity - the opposite of what is observed with loss of consciousness (Luppi et al., 2019). Unlike the other results reported here, the LSD-induced increased in small-world propensity was observed in both integrated and segregated dynamic sub-states (although thresholding the network to only keep the strongest connections changed this behaviour, highlighting the important role of weak edges in the small-world topology of brain networks (Gallos et al., 2012)).

Small-worldness reflects optimal balance of local and global processing in a network (Barttfeld et al., 2015), by combining high local clustering (facilitation sharing of information at a local level) with a short characteristic path length that dramatically reduces the cost of global information transmission. Crucially, the overall increase in small-world propensity observed here arose from the balance of two opposing trends: namely, the deviation of characteristic path length from a corresponding random network (Δ_L_) increased under LSD, reducing the network's small-world propensity; however, this effect was more than compensated by a reduction in the deviation of the network's clustering coefficient from that of a regular network (Δ_C_) – resulting in overall increased small-world propensity. In other words, under the effects of LSD the brain appears to shift the balance of information processing towards a more localised pattern.

These findings are in line with evidence obtained in time-averaged networks after ingestion of ayahuasca (Viol et al., 2017), showing increased clustering coefficient but decreased characteristic path length. However, small-world propensity also exhibited time-specific effects that could not be observed from just considering time-averaged FC: namely, only the increase pertaining to the predominantly integrated state was consistently predictive of subjective experiences induced by LSD: blissful feeling, complex imagery, and ego dissolution. The discovery that LSD-induced ego-dissolution exhibits temporally-specific effects merits special attention because the extent of ego-dissolution experienced during the psychedelic experience has been repeatedly found to predict positive clinical outcomes following psychedelic administration (Griffiths et al., 2016; Majić et al., 2015; Roseman et al., 2018; Ross et al., 2016). Thus, advancing our understanding of the dynamic effects of psychedelics on brain function, and the interaction between brain integration and ego dissolution in particular, may hold further promise for our understanding of the therapeutic effects of psychedelics.

Our results suggest that the predominantly integrated sub-state may be especially relevant for supporting the feeling of integrity of one's sense of self, as indicated by the positive correlation between perturbed balance of local-global processing (quantified by small-world propensity) and subjective feeling of ego dissolution. Indeed, it stands to reason that states of relatively high brain integration should be especially relevant for one's sense of self, when considering the intrinsically integrative nature of the self, which brings together distinct sensory streams into a unified “stream of consciousness” as well as the continuity between past and present.

### 4.1 Limitations

The present study has a number of limitations that should be borne in mind when evaluating its results. Firstly, we acknowledge that the dataset we studied here has been already published on before. As such, it will be an important next step for these results to be replicated in independent datasets and with larger samples, to ensure that our understanding of the effects of LSD on brain function and dynamics is generalisable beyond our limited sample of volunteers. Likewise, it will be important to replicate these results with other serotonergic psychedelics, such as psilocybin or DMT. In the present work, we replicated our results using alternative types and scales of brain parcellation, to ensure their robustness.

It is also worth mentioning that although the study design was placebo-controlled, LSD has profound and clearly apparent subjective effects (as corroborated by the subjective ratings provided by our volunteers) (Carhart-Harris et al., 2016), and as such, it is plausible that volunteers became aware of whether they took LSD or placebo, especially since they were already familiar with the effects of psychedelics. Nevertheless, since this awareness would be driven by the very strong psychological effects of LSD, rather than explicit information about the condition (as would be the case for an open-label design) we consider it unlikely that this may represent a confound for any of our findings.

Although the data was collected using an inert placebo in order to provide a valid baseline while still allowing us to introduce some uncertainty, future studies may benefit from the use of an ‘active placebo’ condition. In particular, contrasting LSD with stimulant drugs that influence arousal levels without producing psychedelic effects may serve multiple purposes: increased effectiveness of the placebo control; a more stringent test of the entropic/anarchic brain hypothesis (Carhart-Harris and Friston, 2019); and also providing a way to control for the effects of arousal on dynamic functional connectivity (Laumann et al., 2017). Indeed, the tangled relationship between brain dynamics, cognition, arousal and its physiological correlates (such as breathing, cardiac and motion changes) is still incompletely understood (Lurie et al., 2018), and as a serotonergic agent, LSD is likely to exert direct or indirect effects across several of these levels. Thus, future studies contrasting LSD with non-psychedelic stimulants may provide an especially promising way to disentangle some of these aspects.

As discussed in previous research using the same dataset (Carhart-Harris et al., 2016), differences in motion under LSD may represent a potential confound, especially in the context of dynamic FC analysis (Laumann et al., 2017). Although global signal regression (GSR) has been proposed as an effective tool to account for potential residual artifacts induced by head motion (Power et al., 2014), in the present study we decided not to include this preprocessing step, in line with our previous work (Carhart-Harris et al., 2016; Luppi et al., 2019). Indeed, the global signal was recently demonstrated to contain relevant information about behaviour and brain topography (Li et al., 2019). Furthermore, GSR mathematically mandates that approximately 50% of correlations will be negative a priori. However, the proportion of anticorrelations has been shown to be biologically relevant, being a robust neural marker of the psychedelic state induced by LSD and other drugs (Carhart-Harris et al., 2016, 2012; Roseman et al., 2014; Tagliazucchi et al., 2016b, 2014) – including in the present study. Use of GSR would obscure this important neurobiological information. Additionally, GSR has been suggested to reduce the test-retest reliability of graph-theoretical metrics (Andellini et al., 2015) and the intersession reliability of dynamic brain state properties (Smith et al., 2018). For all these reasons, instead of GSR we adopted an alternative set of steps to mitigate the potential confound of residual differences in head motion between conditions: by means of the aCompCor denoising method (which is among those recommended for investigations of dynamic connectivity (Lydon-Staley et al., 2019), and has been used before in the context of cartographic profile analysis (Luppi et al., 2019; Shine et al., 2016); the cartographic profile analysis has additionally been demonstrated to be robust to the use of GSR during preprocessing (Shine et al., 2016)); by excluding participants with unacceptably high levels of motion from analyses (Materials and Methods); and by explicitly including mean framewise displacement as a covariate of no interest in our statistical analyses. Finally, we confirmed that our effects of interest did not correlate with mean frame-wise displacement.

Another limitation of the present work, with respect to the relation between functional and structural connectivity, is that the latter was estimated from population-average diffusion imaging templates of the Human Connectome Project (Yeh et al., 2018), rather than being specific for each individual subject, so that the LSD-induced alterations in the correspondence between functional and structural connectivity that we reported here are to be interpreted as deviations from the population average, rather than from the specific individual's anatomical connectome. It is also worth noting that reduced structural-functional similarity was also found during propofol anaesthesia by (Tagliazucchi et al., 2016a). As those authors themselves remarked when relating their results to the opposite ones observed by (Barttfeld et al., 2015), the effect they reported was specific to a set of frontal regions and the thalamus, in contrast with the global effect observed in other reports of structural-functional similarity in anaesthesia, as well as the present study.

Our observation that the proportion of time spent in the primarily integrated sub-state is preserved under LSD may appear to contrast with the recent evidence that a dynamic sub-state of high global synchrony is visited more frequently under the effects of psilocybin (Lord et al., 2019). However, the methodology employed by Lord and colleagues (Lord et al., 2019) was substantially different from the one adopted here: instead of a sliding-windows-based functional connectivity approach, they applied a different technique known as LEiDA (Leading Eigenvector Dynamics Analysis), which captures instantaneous low-dimensional patterns of relative BOLD signal phase-locking (rather than relying on the correlation between BOLD signals across several tens of seconds), subsequently deriving several possible brain sub-states. Because of these substantial methodological differences, it is not obvious how the 7 data-driven sub-states resembling canonical resting-state networks identified by Lord and colleagues would map onto the primarily integrated and segregated sub-states reported here, which were instead identified based on a hypothesis-driven combination of their graph-theoretical properties. Indeed, cartographic profile based on sliding-windows FC and LEiDA are only two among a rapidly expanding number of possible ways to investigate states of dynamic or “time-varying” brain connectivity (Lurie et al., 2018), as well as additional methods to study the dynamics of brain activity rather than connectivity, such as connectome harmonic decomposition (Atasoy et al., 2018, 2017, 2016). Each of these ways of investigating brain dynamics inevitably comes with its own set of strengths and limitations, but as mentioned above when discussing the present results in the context of CHD, converging evidence is already beginning to emerge across different methods.

Finally, studies of the dynamics of brain activity would benefit from a larger number of timepoints and higher temporal resolution, as afforded by EEG and MEG. Likewise, efforts to improve the sampling of subjective experience should be carried out in future work (Petitmengin and Lachaux, 2013), such as through techniques already applied in the study of mind wandering (Christoff et al., 2009). Efforts to more regularly sample subjective experience need to factor in how this will affect spontaneous cognition and brain function. Post-hoc ‘filling in’ of a scanning period based on timestamps and interviewing may provide a novel solution here, as might experience-sampling techniques - alluded to above (Petitmengin and Lachaux, 2013). Further studies could extend the present results by exploiting these complementary methodologies, providing a more direct relation between brain states of predominant integration and segregation, and the subjective experiences induced by LSD and other psychedelics. Higher resolution sampling of the subjective experience may enable us to draw more reliable mappings between epochs of particularly abnormal brain activity and parallel subjective experience. Interactions between brain integration and segregation, complexity, and subjective experience are therefore a particularly appealing area for future research, which may have a bearing on our understanding of the therapeutic mechanisms of psychedelic therapy (Carhart-Harris and Goodwin, 2017).

### 4.2 Conclusion

The main novel finding of the present analysis is that the effects of LSD on brain function and subjective experience are non-uniform in time: rather, they depend on the particular state of the brain at a given point in time. LSD makes globally segregated brain sub-states more complex and more decoupled from structural constraints, as well as reducing functional connectivity of the anterior medial PFC, which is thought to subserve processes of reality monitoring. Thus, in line with the recent REBUS framework (Carhart-Harris and Friston, 2019), LSD may facilitate the exploration of a more diverse repertoire of functional connectivity patterns, specifically by weakening the Bayesian prior on functional connectivity represented by anatomical connections, in concomitance with an attenuation of reality monitoring processes. The predominantly segregated sub-state may be particularly favourable for this effect to take place, in virtue of its enhanced decoupling from anatomical constraints.

Brain network characteristics pertaining to the integrated and segregated sub-states also correlated with different subjective experiences induced by LSD; in particular, ego dissolution was predicted by increased small-world propensity during the predominantly integrated dynamic sub-state. In summary, we have shown that LSD has time-dependent effects on the dynamics of brain function, and may exert its psychedelic effects differently at different points in time, based on the brain's state of integration or segregation.

## Data and code availability statement

Functional MRI data from the original study are available from OpenNeuro: https://openneuro.org/datasets/ds003059/versions/1.0.0. Code for the "cartographic profile" is freely available online (https://github.com/macshine/integration/). Code for the computation of small-world propensity is freely available online (http://www.seas.upenn.edu/~dsb/). The Brain Connectivity Toolbox code used for graph-theoretical analyses is freely available online (https://sites.google.com/site/bctnet/). The CONN toolbox is freely available online (http://www.nitrc.org/projects/conn).

## CRediT authorship contribution statement

**Andrea I. Luppi:** Conceptualization, Methodology, Formal analysis, Writing - original draft, Visualization, Validation. **Robin L. Carhart-Harris:** Investigation, Funding acquisition, Data curation, Writing - review & editing. **Leor Roseman:** Investigation, Data curation. **Ioannis Pappas:** Formal analysis, Data curation. **David K. Menon:** Writing - review & editing, Supervision, Resources. **Emmanuel A. Stamatakis:** Writing - review & editing, Conceptualization, Methodology, Supervision, Project administration, Resources.

## Declaration of Competing Interest

The authors declare no conflict of interest.

## Appendix Supplementary materials

Image, application 1
