# Supplementary material for: LSD alters dynamic integration and segregation in the human brain
Source: Neuroimage. 2021 Feb 15;227:117653. doi: 10.1016/j.neuroimage.2020.117653 (PMC7896102; doi:10.1016/j.neuroimage.2020.117653)
Supplement: Supplementary file 1 [file mmc1.docx]

Supplementary Information for LSD alters dynamic integration and segregation in the human brain

Andrea I. Luppi^a,b^*, Robin L. Carhart-Harris^c^, Leor Roseman^c^, Ioannis Pappas^a,b,d^,

David K. Menon^a,e^, Emmanuel A. Stamatakis^a^

^a^Division of Anaesthesia

^b^Department of Clinical Neurosciences, University of Cambridge, Cambridge, UK

^c^Centre for Psychedelic Research, Department of Brain Sciences, Imperial College

London, London, UK

^d^Department of Psychology, University of California - Berkeley, Berkeley, CA, USA

^e^Wolfson Brain Imaging Centre, University of Cambridge, Cambridge, UK

*Corresponding author

Email address: al857@cam.ac.uk (Andrea I. Luppi)

# Supplementary Materials and Methods

## Subjective Ratings

As reported in the original publication ^1^, VAS subjective ratings were obtained after each scan. The scales included items for intensity, simple imagery, complex imagery, positive mood and ego dissolution and emotional arousal. Specifically, they were phrased as follows: 1) “Please rate the intensity of the drug effects during the last scan”, with a bottom anchor of “no effects”, a mid-point anchor of “moderately intense effects” and a top anchor of “extremely intense effects”; 2) “With eyes closed, I saw patterns and colours”, with a bottom anchor of “no more than usual” and a top anchor of “much more than usual”; 3) “With eyes closed, I saw complex visual imagery”, with the same anchors as item 2; 4) “How positive was your mood for the last scan?”, with the same anchors as item 2, plus a mid-point anchor of “somewhat more than usual”; 5) “I experienced a dissolving of my self or ego”, with the same anchors as item 2; and 6) “Please rate your general level of emotional arousal for the last scan”, with a bottom anchor of “not at all emotionally aroused”, a mid-point anchor of “moderately emotionally aroused” and a top anchor of “extremely emotionally aroused”.

## Cartographic Profile Construction

Following Shine *et al.,* (2016)^2^, Fukushima *et al.,* (2018)^3^, and Luppi *et al.* (2019)^4^, states of higher integration or segregation can be identified from the connectivity between regions, by establishing a “cartographic profile” based on the module assignments of each ROI, considered as a network node. At each time-point, the asymmetric algorithm of Rubinov and Sporns (2011)^5^ implemented in the MATLAB-based Brain Connectivity Toolbox (BCT; [http://www.brain-connectivity-toolbox.net](http://www.brain-connectivity-toolbox.net/))^8^ was used to identify network modules by applying the Louvain greedy algorithm ^6^, which iteratively evaluates different ways of assigning nodes to modules, in order to maximise the resulting modularity function *Q:*

$$Q = \frac{1}{\upsilon^{+}}\sum_{ij} ({w_{ij}}^{+} - {e_{ij}}^{+})\delta_{M_{i}M_{j}}- \frac{1}{\upsilon^{+}+ \upsilon^{-}}({w_{ij}}^{-} - {e_{ij}}^{-})\delta_{M_{i}M_{j}}$$

(S1)

where *υ* is the total weight of the graph (sum of all edges), *w_ij_* is the (signed) weight of the edge between nodes *i* and *j*, *e_ij_* is the weight of an edge divided by the total weight of the graph (positive and negative edges are denoted with ‘+’ and ‘−’ superscripts, respectively), and *δ_MiMj_* is set to 1 when nodes *i* and *j* are in the same module and 0 otherwise.

In the case of signed graphs, a module is defined as a group of nodes that are positively correlated with each other, but negatively correlated with nodes belonging to different modules^7^. Due to its stochastic nature, the algorithm was repeated for 100 iterations for each time- resolved network, and the module size resolution parameter *γ* was set to one, the default^2,3^.

Based on the modularity assignments, we then derived the participation coefficient and within-degree Z-score for each node. The participation coefficient *P_i_* quantifies the degree of connection that a node entertains with nodes belonging to other modules: the more of a node’s connections are towards other modules, the higher its participation coefficient will be^7^.

Conversely, the participation coefficient of a node will be zero if its connections are all with nodes belonging to its own module.

$$P_{i}= 1 - \sum_{s=1}^{M} \left( \frac{\kappa_{is}}{k_{i}} \right)^{2}$$

(S2)

Here, *κ_is_* is the strength of positive connections between node *i* and other nodes in module *s*, *k_i_* is the strength of all its positive connections, and *M* is the number of modules in the network, as identified by a given modularity detection algorithm. The participation coefficient ranges between zero (no connections with other modules) and one (equal connections to all other modules). A network with high average participation coefficient can be expected to have high levels of integration between its constituent modules.

Conversely, the within-module degree Z-score *Z_i_* is a measure of a node’s connectivity with other nodes belonging to its module. It indicates how much larger (or smaller) the node’s connections to other nodes in the module are, relative to the average connection strength within that module. A node with high within-module degree Z-score has stronger-than-average coupling with the other nodes in its module^7^.

$$z_{i} = \frac{\kappa_{is} - \bar{\kappa}_{is}}{\sigma_{\kappa_{is}}}$$

(S3)

where *κ_is_* is the strength of connections between node *i* and other nodes in module *s*, and $\bar{\kappa}$*_is_* and *σ_κis_* are respectively the average and the standard deviation of *κ_is_* over all nodes belonging to module *s*. The Brain Connectivity Toolbox was used to derive both the participation coefficient and within-degree Z-score for each node.

Subsequently, joint histograms of participation coefficient and within-module Z-score were produced for each timepoint^2^, (using MATLAB code made freely available by Shine et al. (2016) at https://github.com/macshine/integration/)^2^, since together, these two measures quantify both a node’s *inter* modular and *intra*modular connectivity. For each subject, the joint patterns were then used to assign each timepoint to one of two clusters, using an unsupervised machine learning algorithm known as k-means clustering (setting *k* = 2)^2^. To avoid the possibility of the algorithm becoming stuck in local minima, it was repeated 500 times with random re-initialisation of the two clusters’ initial points. This was performed individually for each subject and condition. Following Shine *et al* (2016), Pearson correlation was chosen as distance metric for the algorithm. Although based on previous work our interest was a priori on clustering with *k* = 2, the clustering process was also repeated with values of *k* ranging between 2 and 7, using MATLAB’s *evalclusters* function to determine the goodness of different clustering solutions using the Silhouette criterion (Supplementary Figure 1). All of these procedures are as described in our previous work^4^.

Finally, for our clustering of interest (with *k* =2) the cluster with higher mean participation coefficient was labelled as the integrated state, while the cluster with lower average participation coefficient was considered to be the segregated state^2^. For each subject, a centroid matrix of functional connectivity was computed for each state, as the element-wise median of the timepoint-specific FC matrices assigned to the cluster corresponding to that state.

The proportion of time spent in each state was also estimated, as the number of timepoints assigned to that cluster, over the total number of timepoints.

## Structural Connectivity from Human Connectome Project Data

### Human Structural Connectome Template

Diffusion-weighted imaging was not acquired as part of this study; therefore, to obtain an estimate of the anatomical connectivity between brain regions in the healthy population, we relied on the Human Connectome Project (HCP, <http://www.humanconnectome.org/>), population-average template constructed and made publicly available by Yeh and colleagues^8^. The HCP group-average template was constructed from a total of 1021 subjects' diffusion MRI data from the Human Connectome Project (2017 Q4, 1200-subject release^9^). As reported in these studies, a multishell diffusion scheme was used, and the b-values were 1000, 2000, 3000 s/mm2. The number of diffusion sampling directions were 90, 90, and 90, respectively. The in-plane resolution was 1.25 mm. The slice thickness was 1.25 mm.

Following previous work^10^: “The diffusion data were reconstructed in the MNI space using QSDR^11^ to obtain the spin distribution function^12^. QSDR is a model-free method that calculates the orientational distribution of the density of diffusing water in a standard space, to conserve the diffusible spins and preserve the continuity of fiber geometry for fiber tracking. QSDR first reconstructs diffusion-weighted images in native space and computes the quantitative anisotropy (QA) in each voxel. These QA values are used to warp the brain to a template QA volume in Montreal Neurological Institute (MNI) space using a nonlinear registration algorithm implemented in the statistical parametric mapping (SPM) software. A diffusion sampling length ratio of 2.5 was used, and the output resolution was 1 mm. The analysis was conducted using DSI Studio (<http://dsi-studio.labsolver.org>). A modified FACT algorithm^13^ was then used to perform deterministic fiber tracking on the reconstructed group-average data, with the following parameters. Angular cutoff of 55◦, step size of 1.0 mm, minimum length of 10 mm, maximum length of 400mm, spin density function smoothing of 0.0, and a QA threshold determined by DWI signal in the colony-stimulating factor. Each of the streamlines generated was automatically screened for its termination location. A white matter mask was created by applying DSI Studio's default anisotropy threshold (0.6 Otsu's threshold) to the SDF's anisotropy values. The mask was used to eliminate streamlines with premature termination in the white matter region. Deterministic fiber tracking was performed until 1,000,000 streamlines were reconstructed for each individual”. These procedures followed a recently described pipeline^10^.

### Structural Connectivity Matrix Construction

Based on this reconstructed group-average tractogram, DTI data were parcellated according to each of the three parcellation schemes considered here. Then, a binary and undirected connectivity matrix *S* was derived by setting entry *S_ij_* = 1 if there were white matter streamlines connecting regions *i* and *j* end-to-end, and zero otherwise.

## Graph-Theoretical Measures

### Characteristic Path Length

Characteristic path length (*L*) is a network-wide measure of how effortful it is on average to move between different nodes in the network. This metric is calculated as the average length of the shortest path *d* between every pair of nodes in the network.

$$L = \frac{1}{n} \sum_{i}^{n} \frac{\sum_{j\neq i}^{n} \left( d_{ij} \right)}{n-1}$$

(S4)

The shortest path length between two nodes *i* and *j*, *d_ij_,* represents the effort required to move between them, such that smaller values indicate less effort/easier communication. Different networks may attach different meaning to the quantity being minimised, such as time in a transport network, or cost. In the simplified case of a binarised network, the shortest path (geodesic distance) between two nodes *i* and *j* is the smallest number of edges that need to be traversed to move from *i* to *j*. When considering a weighted network, a stronger edge is understood as implying easier communication between the two nodes (analogous to how large a road is: larger roads make communication easier). Thus, in weighted graphs the shortest path d between *i* and *j* is calculated as the minimum sum of inverse of the edge weights that need to be traversed between the two nodes. Thus, the characteristic path length is understood as inversely related to the capacity for global processing across the whole network^7^.

### Clustering Coefficient

The clustering coefficient of node *i* (*C_i_*) is a node-specific measure of how well connected a node’s neighbourhood is; in a binarized graph, it is calculated as the fraction of neighbours of the node that are also neighbours of each other.

$$C_{i} =\frac{{2t}_{i}}{k_{i}(k_{i} - 1)}$$

(S5)

where *t_i_* is the number of triangles around node *i,*and $k_{i}$ is the number of edges connected to node *i.*

Following Muldoon *et al* (2016), for weighted graphs we adopt the generalisation of the clustering coefficient based on subgraph intensity, proposed by Onnela *et al* (2005)^14^:

$$C_{i} =\frac{1}{k_{i}(k_{i} - 1)}\sum_{j,k}^{n} \left( \hat{w}_{ij} + \hat{w}_{jk} + \hat{w}_{ik} \right)^{1/3}$$

(S6)

With *w_ij_* being the strength of a connection between nodes *i* and *j*, and $\hat{w}_{ij}=$*w_ij_* / max(*w*).

The mean of all nodes’ clustering coefficients (i.e. the network’s mean clustering coefficient, *C*), indicates how well connected, on average, the neighbourhoods present in the network tend to be. When applied to brain networks, the clustering coefficient is thought to represent the degree of information integration at a local level, and hence the potential for efficiently performing specialised local processing^7^.

# Supplementary Figures


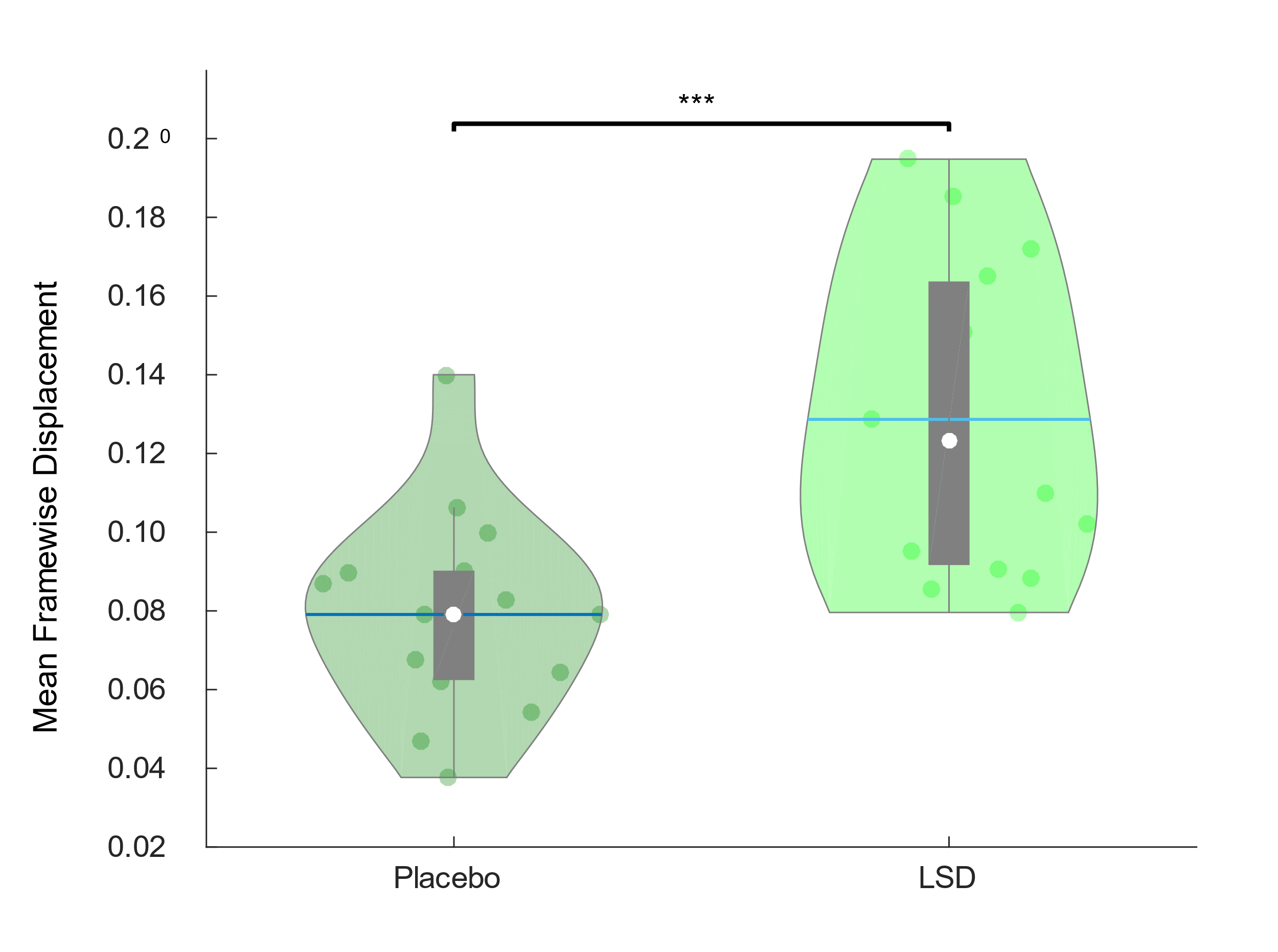


**Supplementary Figure 1.** Differences in head motion (mean framewise displacement) between placebo and LSD. Violin plots indicate the distribution of participants in each condition (coloured circles). White circle, mean; blue center line, median; box limits, upper and lower quartiles; whiskers, 1.5x interquartile range.

**
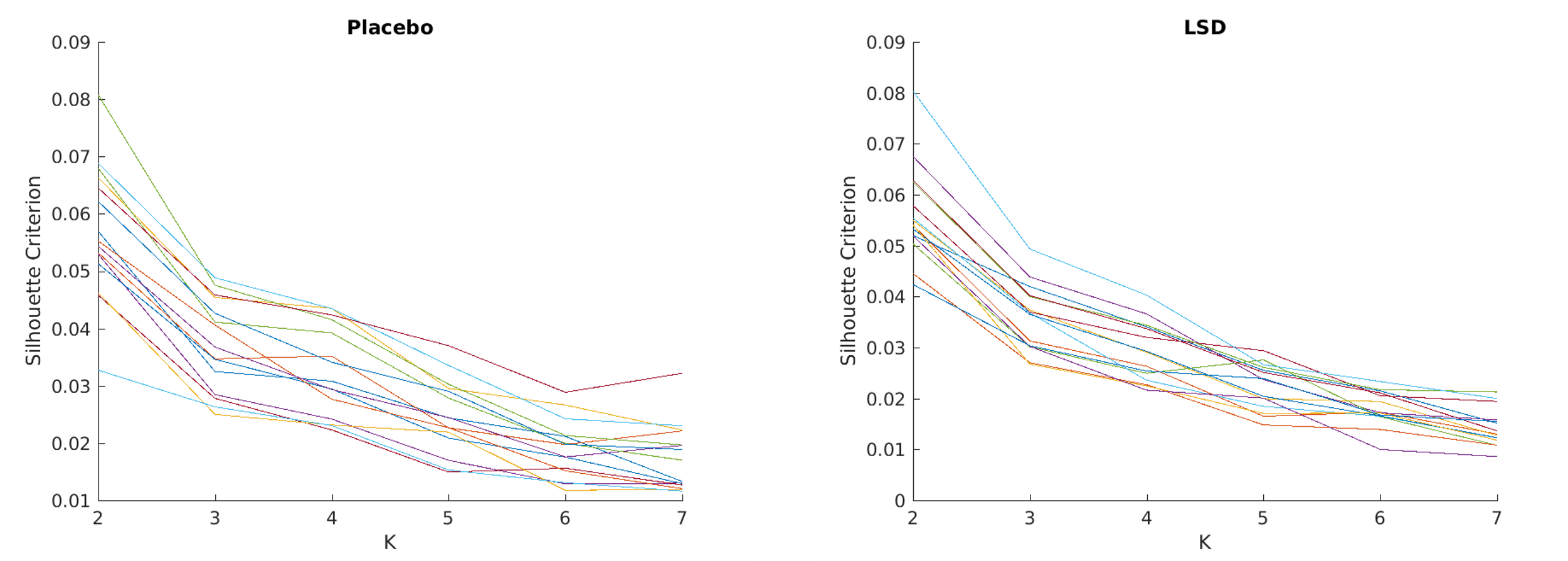
**

**Supplementary Figure 2.** Silhouette criterion for the choice of best number of clusters K for each participant, separately for placebo and LSD. In each case, K = 2 is the most appropriate number of clusters.


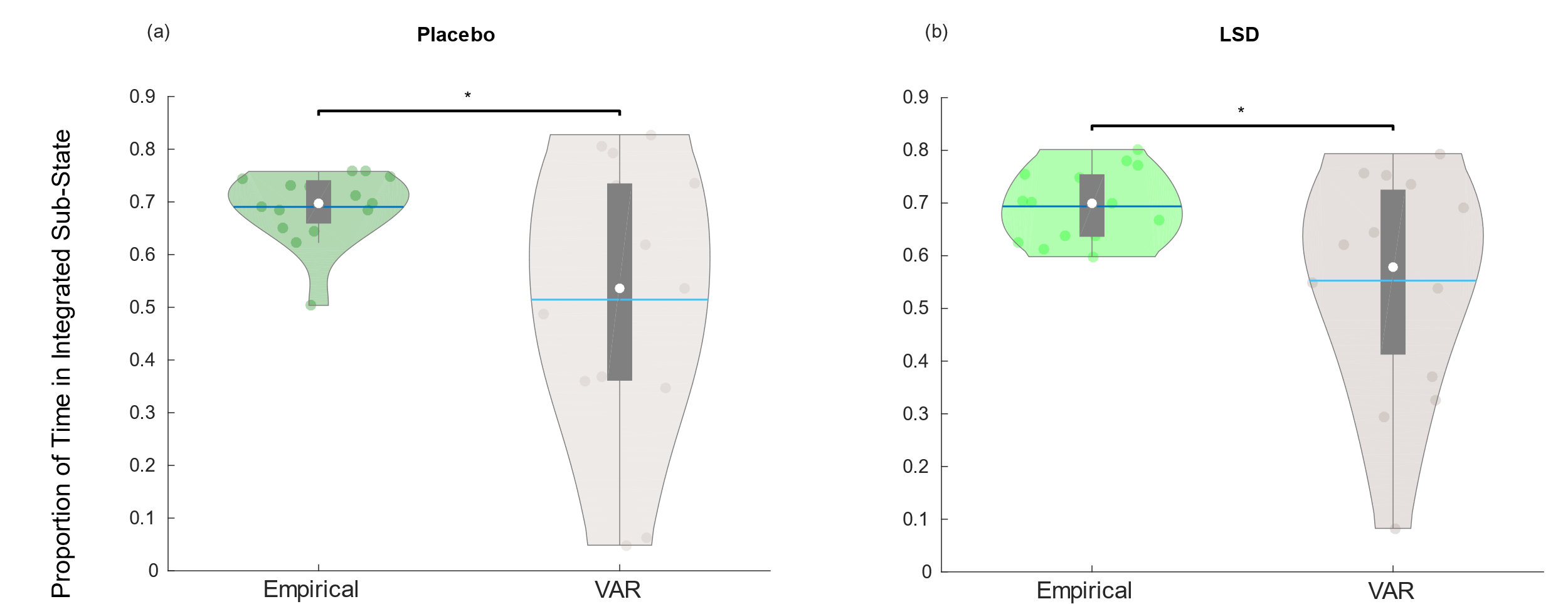


**Supplementary Figure 3.** Proportion of time spent in the predominantly integrated sub-state is significantly higher in empirical data than surrogate stationary data obtained from a VAR model, for both placebo (a) and LSD (b). Violin plots indicate the distribution of participants in each condition (coloured circles). White circle, mean; blue center line, median; box limits, upper and lower quartiles; whiskers, 1.5x interquartile range.


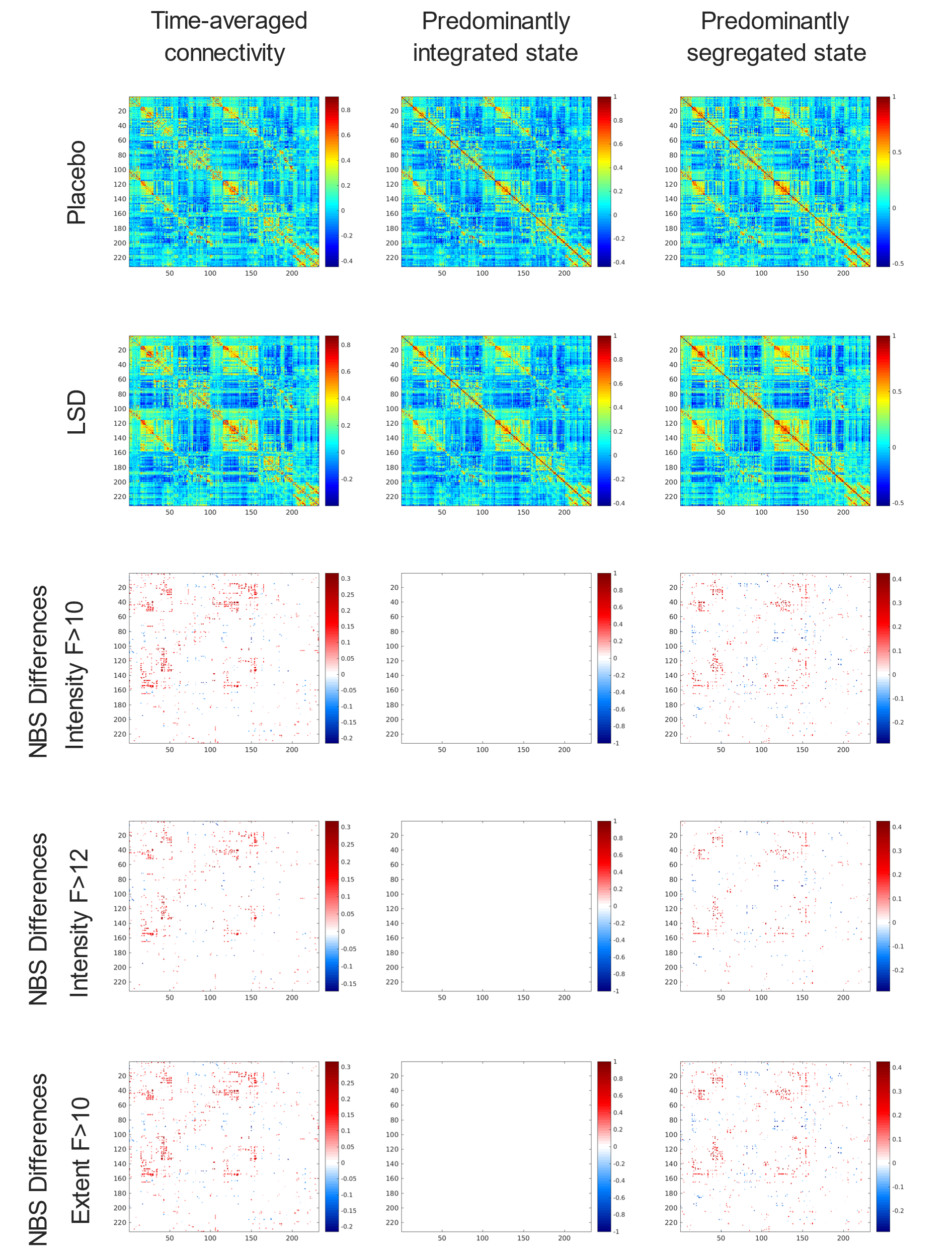


**Supplementary Figure 4.** Matrices of functional connectivity (Pearson correlation) for the augmented Schaefer-232 atlas, for the time-averaged connectivity, and for the primarily integrated and segregated sub-states. The first two rows indicate group-averaged FC for placebo and LSD. The last three rows show significant differences between the two conditions, as detected by the Network Based Statistic with different choices of threshold (intensity-based F-threshold of 10, intensity-based F-threshold of 12, and extent-based F-threshold of 10) (note that no significant differences were observed for the integrated sub-state). Here, red indicates LSD > placebo, and blue indicates placebo > LSD.


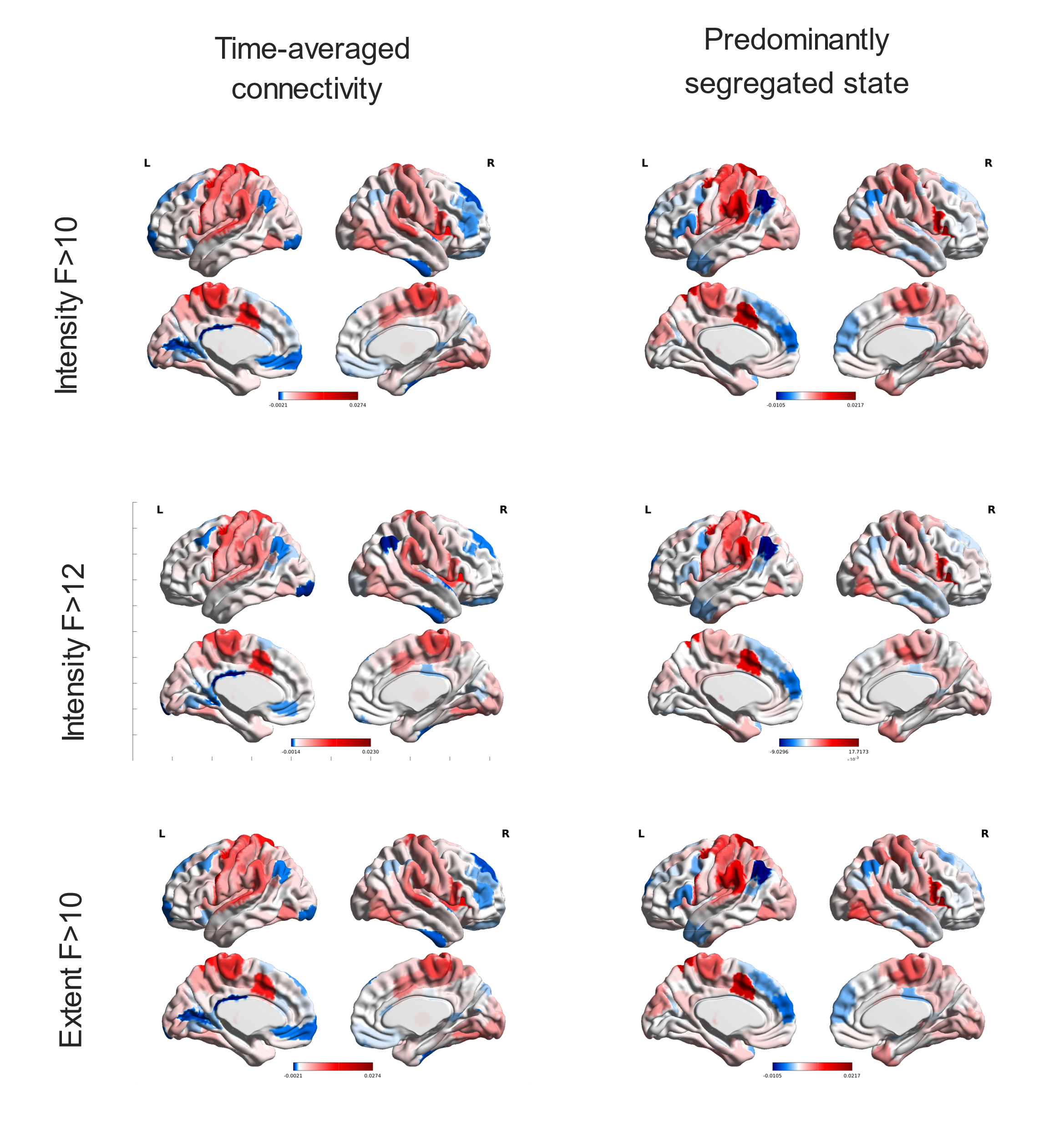


**Supplementary Figure 5.** Surface projections of the total change in connectivity (sum of significant connection changes) for each region of the augmented Schaefer-232 atlas (bottom), for time-averaged functional connectivity (left) and the predominantly segregated sub-state (right), according to different ways of selecting the Network Based Statistic threshold. Note that no significant differences were detected in the predominantly integrated sub-state.


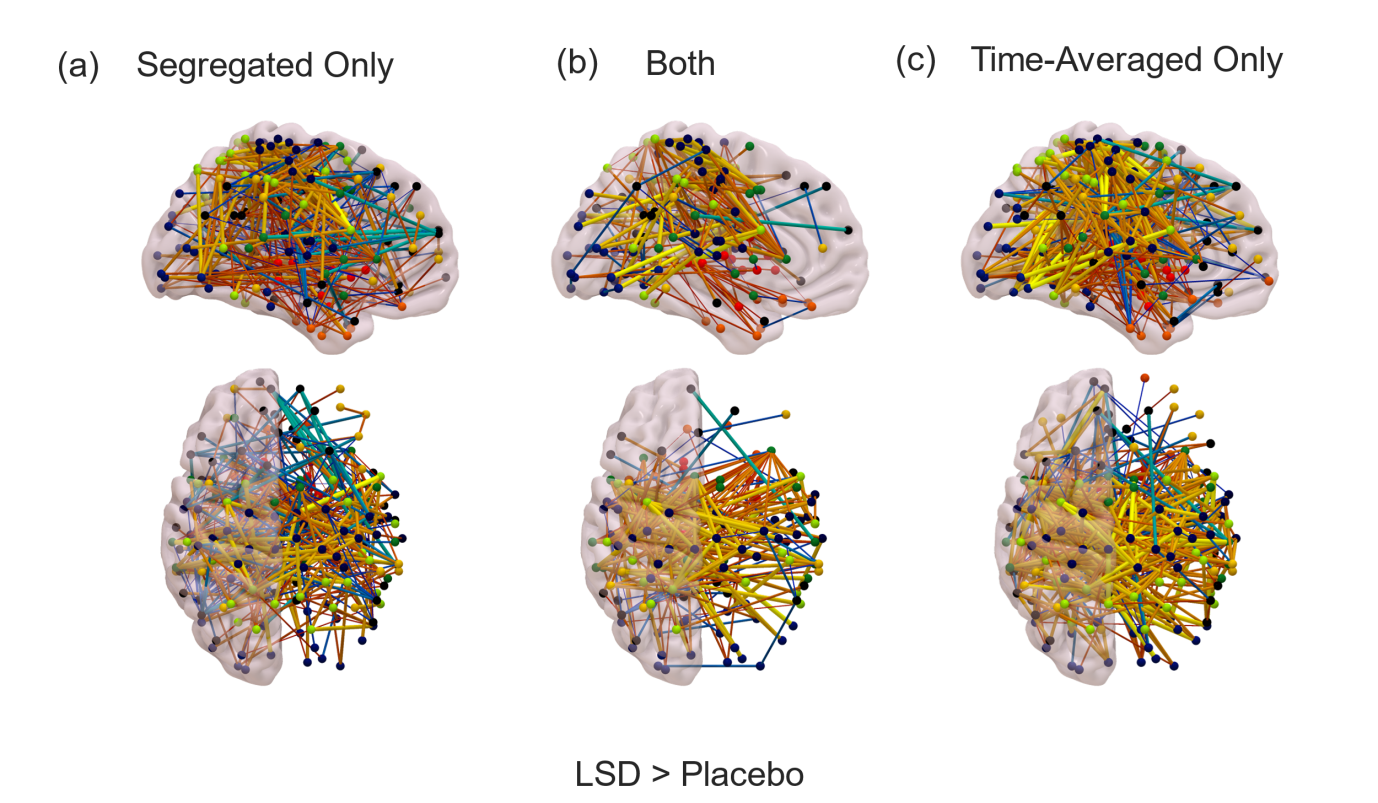


**Supplementary Figure 6.** Brain networks of significant differences in functional connectivity between placebo and LSD. (a) Significantly different edges that are only found in the predominantly segregated dynamic sub-state. (b) Significantly different edges that are found in both the predominantly segregated dynamic sub-state, and in time-averaged functional connectivity. (c) Significantly different edges that are only found in time-averaged functional connectivity. Statistical significance was determined by the Network Based Statistic with an intensity-based F-threshold of 10 (note that no significant differences were observed for the integrated sub-state). Edges between nodes of the augmented Schaefer-232 atlas are shown on the sagittal (top) and axial (bottom) planes, in neurological convention (L is L). Here, yellow indicates LSD > placebo, and blue indicates placebo > LSD.


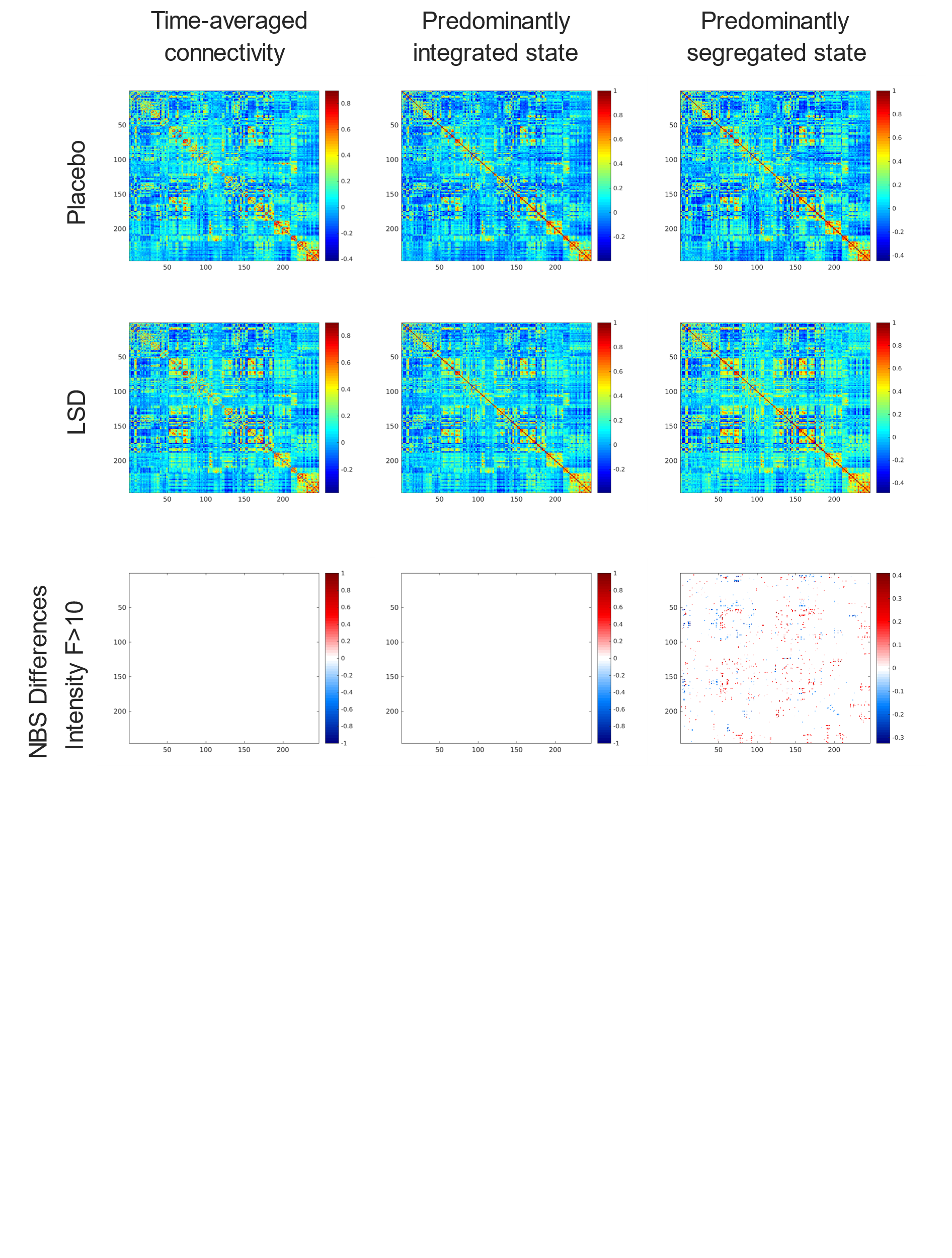


**Supplementary Figure 7.** Matrices of functional connectivity (Pearson correlation) for the 246-ROI Brainnetome atlas, for the time-averaged connectivity, and for the primarily integrated and segregated sub-states. The first two rows indicate group-averaged FC for placebo and LSD. The last row shows significant differences between the two conditions, as detected by the Network Based Statistic with an intensity-based F-threshold of 10. Here, red indicates LSD > placebo, and blue indicates placebo > LSD.


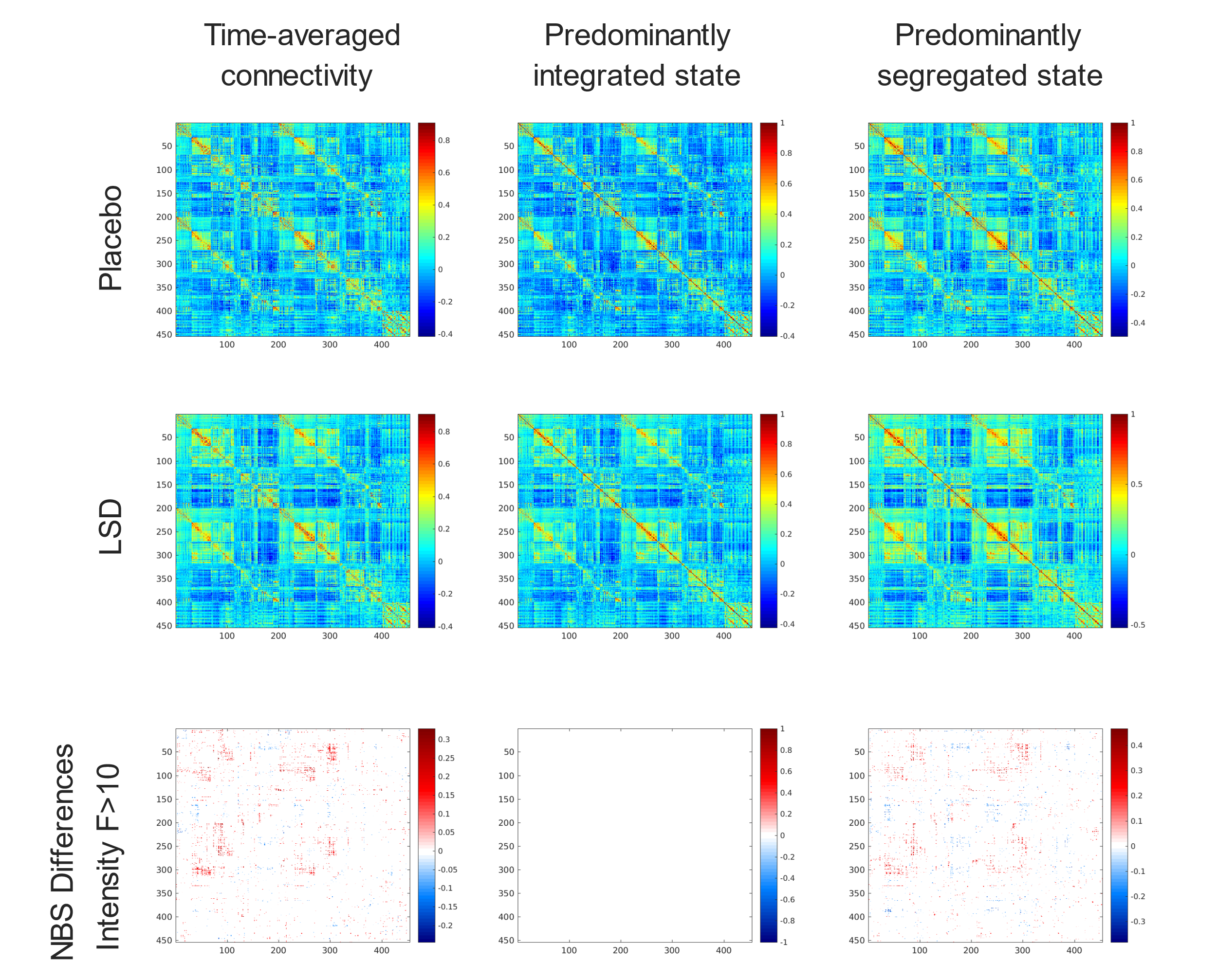


**Supplementary Figure 8.** Matrices of functional connectivity (Pearson correlation) for the augmented Schaefer-454 atlas, for the time-averaged connectivity, and for the primarily integrated and segregated sub-states. The first two rows indicate group-averaged FC for placebo and LSD. The last row shows significant differences between the two conditions, as detected by the Network Based Statistic with an intensity-based F-threshold of 10. Here, red indicates LSD > placebo, and blue indicates placebo > LSD.


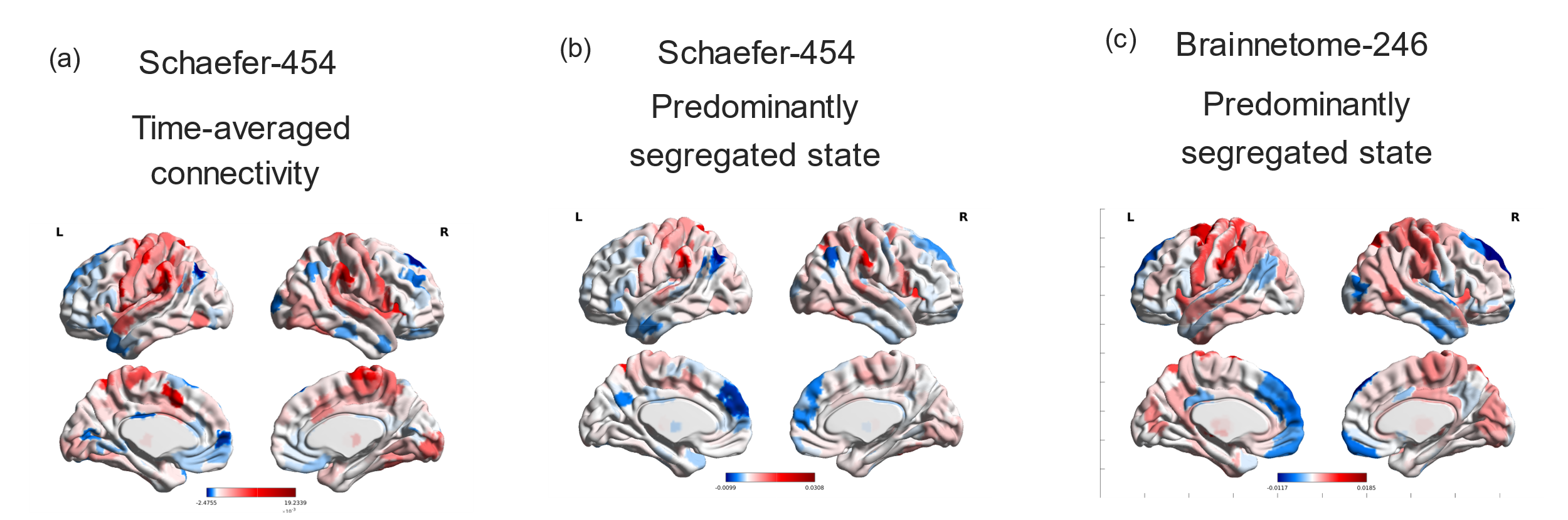


**Supplementary Figure 9.** Surface projection of the total change in connectivity (sum of significant connection changes) for alternative parcellations. (a) Total change in time-averaged functional connectivity for the augmented Schaefer-454 atlas. (b) Total change in connectivity of the predominantly segregated sub-state for the augmented Schaefer-454 atlas. (c) Total change in connectivity of the predominantly segregated sub-state for the Brainnetome-246. Note atlas that no significant differences were detected for time-averaged functional connectivity for the Brainnetome atlas, and no significant differences were detected in either atlas for the predominantly integrated sub-state.


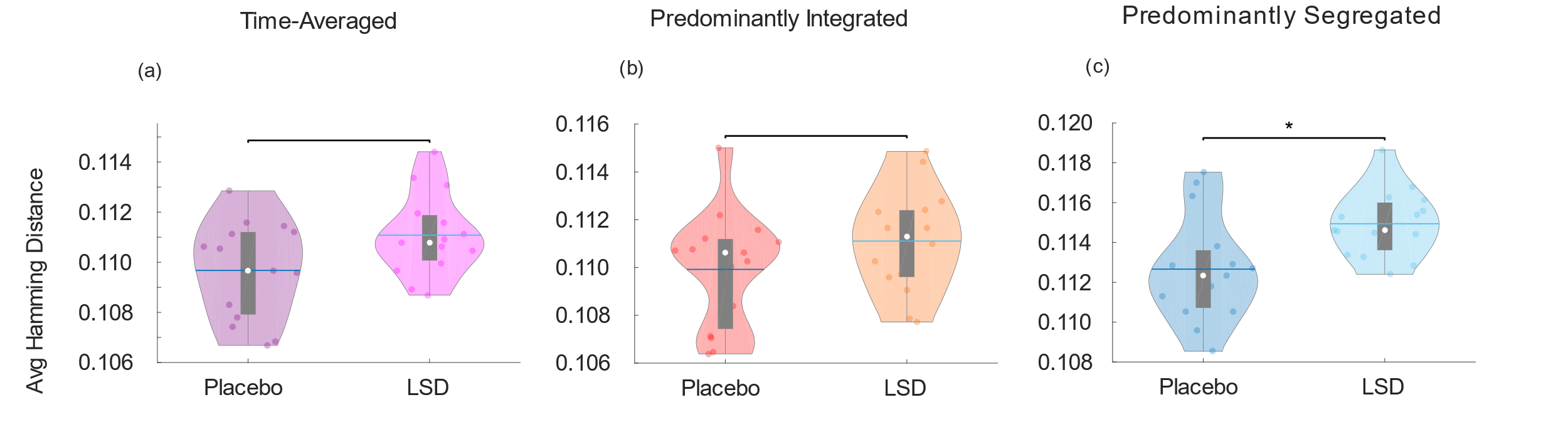
**Supplementary Figure 10.** Average Hamming distance (over all brain regions) between functional and structural connectivity, for time-averaged functional connectivity (a) the predominantly integrated sub-state (b) and the predominantly segregated dynamic sub-state (c). Violin plots indicate the distribution of participants in each condition (coloured circles). White circle, mean; blue center line, median; box limits, upper and lower quartiles; whiskers, 1.5x interquartile range. * p < 0.05.


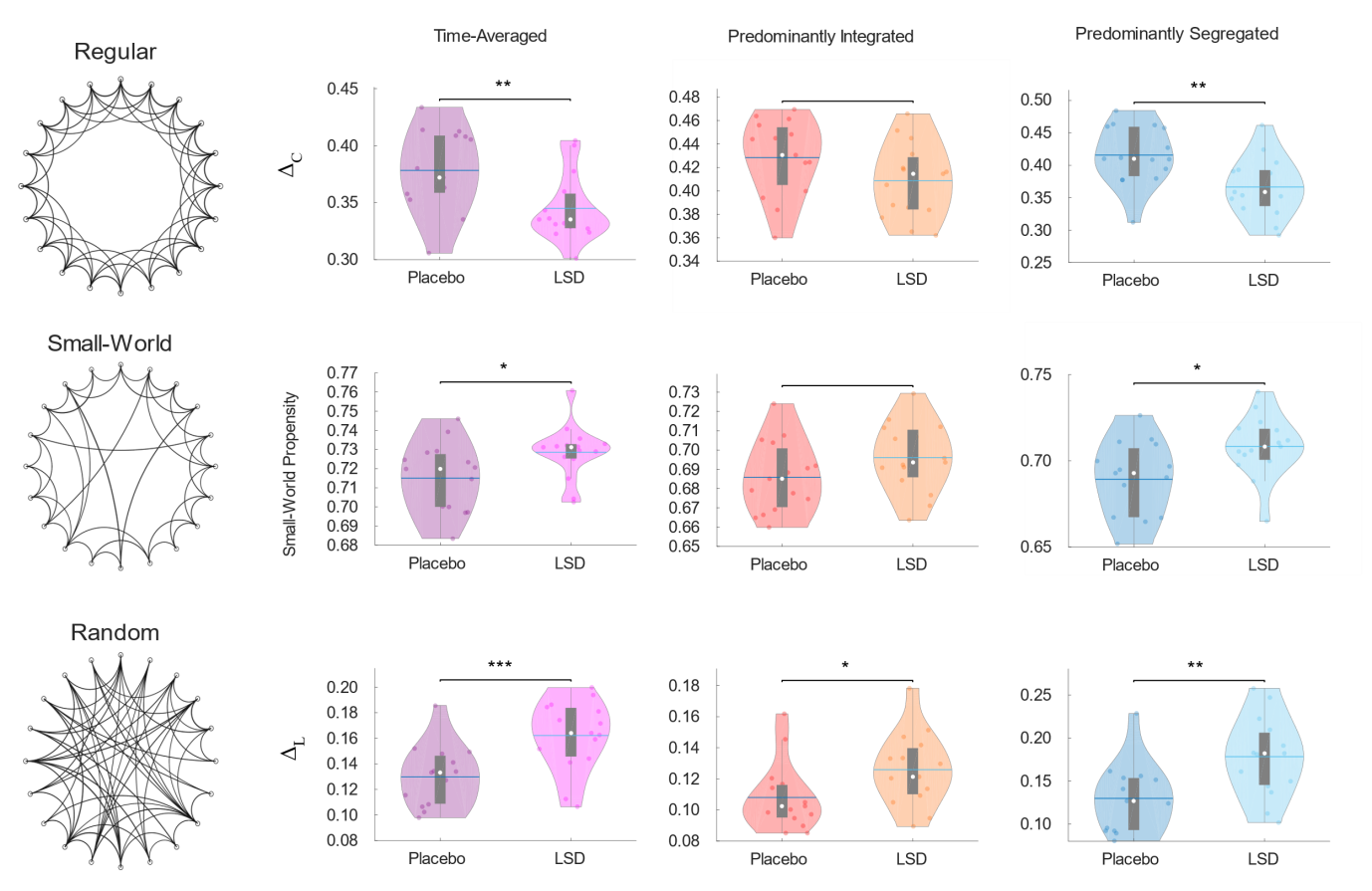


**Supplementary Figure 11. Increased small-world propensity of time-averaged and dynamic binary brain networks under LSD.** Comparison of deviation from a regular network in terms of clustering (Δ_C_; top row), small-world propensity (middle row), and deviation from a random network in terms of characteristic path length (Δ_L_), for binary networks obtained from time-averaged functional connectivity, the predominantly integrated sub-state, and the predominantly segregated dynamic sub-state. Networks were obtained by thresholding the corresponding FC matrices at density of 10, 15, 20 and 25%, and the resulting values were averaged within each participant before analysis. Violin plots show the distribution of H-index values for each group. White circle, mean; blue center line, median; box limits, upper and lower quartiles; whiskers, 1.5x interquartile range. * *p* < 0.05; ** *p* < 0.01; *** *p* < 0.001.


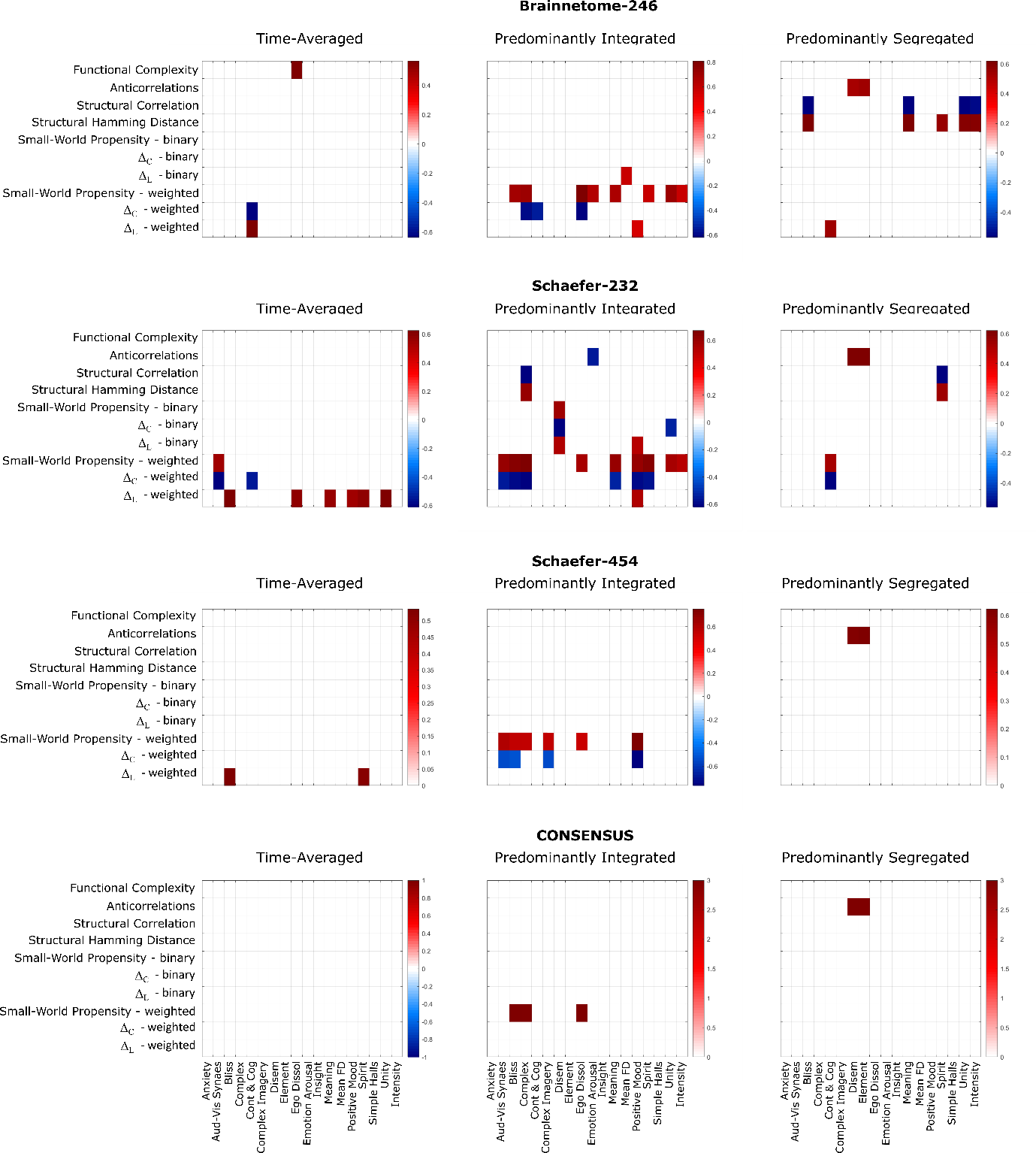


**Supplementary Figure 12.** Significant correlations (Spearman’s rho) between deltas (LSD – Placebo) in subjective ratings (plus motion) and brain measures, for the time-averaged (left column), predominantly integrated dynamic sub-state (middle column) and predominantly segregated dynamic sub-state (right column). Top row: measures are obtained from the 246-region Brainnetome atlas. Second row: measures are obtained from the 232-region augmented Schaefer atlas. Third row: measures are obtained from the 454-region augmented Schaefer atlas. Bottom row: colored cells indicate correlations that are significant with all three parcellations. Non-significant correlations (*p <* 0.05) are not shown. Anxiety: ASC anxiety score; Aud-Vis Synaes: ASC audio-visual synaesthesia score; Bliss: ASC feeling of blissfulness score; Complex: ASC complex imagery score; Complex Imagery: VAS complex imagery score; Disem: ASC feeling of disembodiment score; Element: ASC elementary imagery score; Ego Dissol: VAS ego-dissolution score; Emotion Arousal: VAS emotional arousal score; Insight: ASC feeling of insight score; Meaning: ASC feeling of meaningfulness score; Mean FD: mean frame-wise displacement measure of in-scanner head motion; Positive Mood: VAS rating of positive mood; Spirit: ASC score of spiritual experience; Simple Halls: VAS score of simple hallucinations; Unity: ASC rating for the experience of unity; Intensity: VAS score for the overall intensity of the subjective experience.

# Supplementary Tables

**Supplementary Table 1. Statistical estimates of the comparison between LSD and placebo, for time-averaged connectivity and for the predominantly integrated and segregated dynamic sub-states, using the Brainnetome-246 parcellation**

| Measure | Estimate | SE | tStat | pValue | Sig |
| --- | --- | --- | --- | --- | --- |
| Time-Averaged Functional Connectivity | | | | |  |
| Functional Complexity | 0.038 | 0.018 | 2.154 | 0.040 | * |
| Proportion of Anticorrelations | -0.060 | 0.027 | -2.233 | 0.034 | * |
| Structural-Functional Correlation | -0.007 | 0.006 | -1.297 | 0.205 | - |
| Average Structural-Functional Hamming Distance | 0.001 | 0.001 | 1.344 | 0.190 | - |
| Small-World Propensity - Binary | 0.016 | 0.007 | 2.219 | 0.035 | * |
| ΔC - Binary | -0.037 | 0.015 | -2.531 | 0.017 | * |
| ΔL – Binary | 0.032 | 0.010 | 3.292 | 0.003 | ** |
| Small-World Propensity - Weighted | 0.050 | 0.016 | 3.225 | 0.003 | ** |
| ΔC - Weighted | -0.105 | 0.036 | -2.897 | 0.007 | ** |
| ΔL – Weighted | 0.035 | 0.013 | 2.561 | 0.016 | * |
| Predominantly Integrated Dynamic Sub-State | | | | |  |
| Functional Complexity | 0.037 | 0.023 | 1.622 | 0.116 | - |
| Proportion of Anticorrelations | -0.058 | 0.032 | -1.828 | 0.079 | - |
| Structural-Functional Correlation | -0.005 | 0.006 | -0.772 | 0.447 | - |
| Average Structural-Functional Hamming Distance | 0.001 | 0.001 | 0.800 | 0.430 | - |
| Small-World Propensity - Binary | 0.012 | 0.009 | 1.326 | 0.196 | - |
| ΔC - Binary | -0.024 | 0.014 | -1.707 | 0.099 | - |
| ΔL – Binary | 0.017 | 0.008 | 2.238 | 0.034 | * |
| Small-World Propensity - Weighted | 0.041 | 0.020 | 2.068 | 0.048 | * |
| ΔC - Weighted | -0.072 | 0.038 | -1.894 | 0.069 | - |
| ΔL – Weighted | 0.025 | 0.013 | 1.861 | 0.074 | - |
| Predominantly Segregated Dynamic Sub-State | | | | |  |
| Functional Complexity | 0.046 | 0.017 | 2.742 | 0.011 | * |
| Proportion of Anticorrelations | -0.039 | 0.019 | -2.055 | 0.050 | * |
| Structural-Functional Correlation | -0.018 | 0.008 | -2.421 | 0.022 | * |
| Average Structural-Functional Hamming Distance | 0.002 | 0.001 | 2.429 | 0.022 | * |
| Small-World Propensity - Binary | 0.033 | 0.010 | 3.231 | 0.003 | ** |
| ΔC - Binary | -0.071 | 0.020 | -3.507 | 0.002 | ** |
| ΔL – Binary | 0.058 | 0.016 | 3.701 | 0.001 | *** |
| Small-World Propensity - Weighted | 0.064 | 0.018 | 3.455 | 0.002 | ** |
| ΔC - Weighted | -0.141 | 0.038 | -3.668 | 0.001 | ** |
| ΔL – Weighted | 0.053 | 0.014 | 3.677 | 0.001 | ** |
| Integration-Segregation | | | | |  |
| Integration-Segregation Temporal Entropy | -0.001 | 0.025 | -0.056 | 0.956 | - |
| Proportion of Time in Integrated State | -0.001 | 0.039 | -0.036 | 0.971 | - |

Estimate, estimated difference between conditions; SE, standard error; pValue, p-value; tStat, test statistic; * *p* < 0.05; ** *p* < 0.01 *** *p* < 0.001.

**Supplementary Table 2. Statistical estimates of the comparison between LSD and placebo for time-averaged FC and for the predominantly integrated and segregated dynamic sub-states, using the Schaefer-454 parcellation**

| Measure | Estimate | SE | tStat | pValue | Sig |
| --- | --- | --- | --- | --- | --- |
| Time-Averaged Functional Connectivity | | | | |  |
| Functional Complexity | 0.036 | 0.017 | 2.123 | 0.043 | * |
| Proportion of Anticorrelations | -0.051 | 0.023 | -2.205 | 0.036 | * |
| Structural-Functional Correlation | -0.013 | 0.005 | -2.474 | 0.020 | * |
| Average Structural-Functional Hamming Distance | 0.001 | 0.000 | 2.466 | 0.020 | * |
| Small-World Propensity - Binary | 0.026 | 0.010 | 2.485 | 0.019 | * |
| ΔC - Binary | -0.048 | 0.018 | -2.696 | 0.012 | ** |
| ΔL – Binary | 0.036 | 0.010 | 3.443 | 0.002 | ** |
| Small-World Propensity - Weighted | 0.057 | 0.017 | 3.407 | 0.002 | ** |
| ΔC - Weighted | -0.114 | 0.034 | -3.386 | 0.002 | ** |
| ΔL – Weighted | 0.037 | 0.012 | 3.003 | 0.006 | ** |
| Predominantly Integrated Dynamic Sub-State | | | | |  |
| Functional Complexity | 0.031 | 0.018 | 1.720 | 0.097 | - |
| Proportion of Anticorrelations | -0.047 | 0.026 | -1.826 | 0.079 | - |
| Structural-Functional Correlation | -0.011 | 0.006 | -1.658 | 0.109 | - |
| Average Structural-Functional Hamming Distance | 0.001 | 0.000 | 1.644 | 0.112 | - |
| Small-World Propensity - Binary | 0.018 | 0.011 | 1.679 | 0.105 | - |
| ΔC - Binary | -0.030 | 0.017 | -1.775 | 0.087 | - |
| ΔL – Binary | 0.018 | 0.008 | 2.294 | 0.030 | * |
| Small-World Propensity - Weighted | 0.039 | 0.018 | 2.132 | 0.042 | * |
| ΔC - Weighted | -0.071 | 0.032 | -2.180 | 0.038 | * |
| ΔL – Weighted | 0.024 | 0.011 | 2.195 | 0.037 | * |
| Predominantly Segregated Dynamic Sub-State | | | | |  |
| Functional Complexity | 0.046 | 0.019 | 2.460 | 0.021 | * |
| Proportion of Anticorrelations | -0.040 | 0.015 | -2.654 | 0.013 | * |
| Structural-Functional Correlation | -0.020 | 0.006 | -3.324 | 0.003 | ** |
| Average Structural-Functional Hamming Distance | 0.001 | 0.000 | 3.298 | 0.003 | ** |
| Small-World Propensity - Binary | 0.034 | 0.014 | 2.359 | 0.026 | * |
| ΔC - Binary | -0.066 | 0.025 | -2.618 | 0.014 | * |
| ΔL – Binary | 0.055 | 0.017 | 3.270 | 0.003 | ** |
| Small-World Propensity - Weighted | 0.069 | 0.021 | 3.356 | 0.002 | ** |
| ΔC - Weighted | -0.151 | 0.044 | -3.465 | 0.002 | ** |
| ΔL – Weighted | 0.053 | 0.016 | 3.305 | 0.003 | ** |
| Integration-Segregation | | | | |  |
| Integration-Segregation Temporal Entropy | -0.012 | 0.029 | -0.421 | 0.677 | - |
| Proportion of Time in Integrated State | 0.012 | 0.030 | 0.394 | 0.697 | - |

Estimate, estimated difference between conditions; SE, standard error; pValue, p-value; tStat, test statistic; * *p* < 0.05; ** *p* < 0.01 *** *p* < 0.001.

# Supplementary References

1. Carhart-Harris, R. L., Muthukumaraswamy, S., Roseman, L., Kaelen, M., Droog, W., Murphy, K., Tagliazucchi, E., Schenberg, E. E., Nest, T., Orban, C., Leech, R., Williams, L. T., Williams, T. M., Bolstridge, M., Sessa, B., McGonigle, J., Sereno, M. I., Nichols, D., Hellyer, P. J., *et al.* Neural correlates of the LSD experience revealed by multimodal neuroimaging. *Proc. Natl. Acad. Sci.* **113**, 201518377 (2016).

2. Shine, J. M., Bissett, P. G., Bell, P. T., Koyejo, O., Balsters, J. H., Gorgolewski, K. J., Moodie, C. A. & Poldrack, R. A. The Dynamics of Functional Brain Networks: Integrated Network States during Cognitive Task Performance. *Neuron* **92**, 544–554 (2016).

3. Fukushima, M., Betzel, R. F., He, Y., van den Heuvel, M. P., Zuo, X. N. & Sporns, O. Structure–function relationships during segregated and integrated network states of human brain functional connectivity. *Brain Struct. Funct.* **223**, 1091–1106 (2018).

4. Luppi, A. I., Craig, M. M., Finoia, P., Williams, G. B., Naci, L., Menon, D. K. & Emmanuel, A. Consciousness-specific dynamic interactions of brain integration and functional diversity. *Nat. Commun.* (2019).

5. Rubinov, M. & Sporns, O. Weight-conserving characterization of complex functional brain networks. *Neuroimage* **56**, 2068–2079 (2011).

6. Blondel, V. D., Guillaume, J.-L., Lambiotte, R. & Lefebvre, E. Fast unfolding of communities in large networks. *J. Stat. Mech. Theory Exp.* **2008**, P10008 (2008).

7. Rubinov, M. & Sporns, O. Complex network measures of brain connectivity: Uses and interpretations. *Neuroimage* **52**, 1059–1069 (2010).

8. Yeh, F.-C., Panesar, S., Fernandes, D., Meola, A., Yoshino, M., Fernandez-Miranda, J. C., Vettel, J. M. & Verstynen, T. Population-averaged atlas of the macroscale human structural connectome and its network topology. *Neuroimage* **178**, 57–68 (2018).

9. Van Essen, D. C., Smith, S. M., Barch, D. M., Behrens, T. E. J., Yacoub, E. & Ugurbil, K. The WU-Minn Human Connectome Project: An overview. *Neuroimage* **80**, 62–79 (2013).

10. Luppi, A. I. & Stamatakis, E. A. Combining Network Topology and Information Theory to Construct Representative Brain Networks. *Netw. Neurosci.* (2020).

11. Yeh, F.-C., Wedeen, V. J. & Tseng, W.-Y. I. Estimation of fiber orientation and spin density distribution by diffusion deconvolution. *Neuroimage* **55**, 1054–1062 (2011).

12. Yeh, F. C., Wedeen, V. J. & Tseng, W. Y. I. Generalized q-sampling imaging. *IEEE Trans. Med. Imaging* **29**, 1626–1635 (2010).

13. Yeh, F.-C., Verstynen, T. D., Wang, Y., Fernández-Miranda, J. C. & Tseng, W.-Y. Deterministic Diffusion Fiber Tracking Improved by Quantitative Anisotropy. *PLoS One* **8**, 80713 (2013).

14. Onnela, J. P., Saramäki, J., Kertész, J. & Kaski, K. Intensity and coherence of motifs in weighted complex networks. *Phys. Rev. E - Stat. Nonlinear, Soft Matter Phys.* **71**, (2005).
